# Enhanced photocatalytic activity in ZnO nanoparticles developed using novel *Lepidagathis ananthapuramensis* leaf extract†

**DOI:** 10.1039/d2ra06967a

**Published:** 2023-01-06

**Authors:** Supin K K, Parvathy Namboothiri P M, M. Vasundhara

**Affiliations:** a Polymers and Functional Materials Department, CSIR- Indian Institute of Chemical Technology Hyderabad 500007 India mvas@iict.res.in; b Academy of Scientific and Innovative Research (AcSIR) Ghaziabad 201002 India

## Abstract

The present study focuses on the green synthesis of zinc oxide nanoparticles (ZnO NPs) using a novel *Lepidagathis ananthapuramensis* (LA) leaf extract and a systematic study on the photocatalytic degradation of methylene blue (MB) dye. The structural, thermal, morphological, optical, and surface area analysis of prepared ZnO NPs were examined using X-ray diffraction (XRD), UV-visible spectroscopy, Raman spectroscopy, Fourier transform infrared spectroscopy (FT-IR), X-ray photoelectron spectroscopy (XPS), Brunauer–Emmett–Teller (BET) analysis, thermogravimetric analysis (TGA), field emission-scanning electron microscopy (FE-SEM), energy dispersive X-ray analysis (EDAX) and high-resolution transmission electron microscopy (HR-TEM). The LA stabilised ZnO NPs produced NPs with diverse morphologies, low band gap and cost-effective high yield of production. A systematic study has been carried out to determine the crystallinity and crystallite size of ZnO NPs based on the concentration of Zn(NO_3_)_2_ precursor, concentration of LA leaf extract, calcination temperature and calcination time. The crystallinity and crystallite size of ZnO NPs were evaluated based on the XRD technique. The photocatalytic activity of ZnO NPs was thoroughly investigated for the degradation of MB dye based on various physicochemical parameters such as reaction time, concentration of catalyst, concentration of precursors, concentration of LA extract, concentration of MB, calcination temperature and calcination time. These systematic photocatalytic studies followed green protocols and provided an excellent photocatalytic efficiency result of 96–98.5% towards the decomposition of MB. Hence, this material can work as a potential candidate for waste water treatment by also degrading other toxic dyes.

## Abstract 2

Focussing on the green synthesis of ZnO NPs using a novel *Lepidagathis ananthapuramensis* (LA) leaf template and its photocatalytic activity.

## Graphical abstract

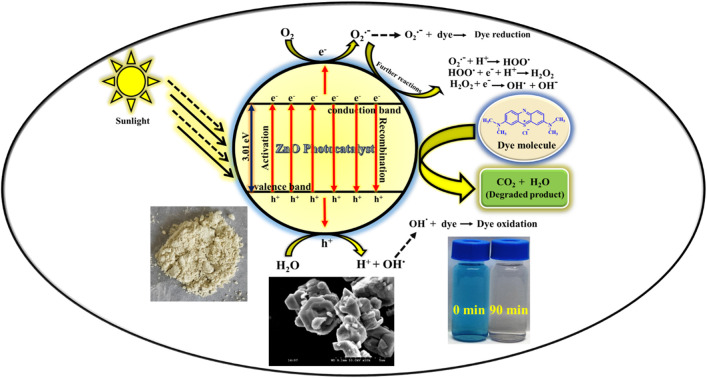

## 1. Introduction

Over the last decade, various metal oxides such as ZnO,^1–3^ CuO,^4^ NiO,^5^ CoO,^6^ Fe_2_O_3_,^7^ MnO_2_,^8^ TiO_2_,^9,10^ SnO_2_,^11^ MgO,^12^*etc* have been synthesised for numerous applications, among which photocatalytic applications and waste water treatment have been explored enormously. Among various metal oxides, ZnO and TiO_2_ NPs are studied the most due to their non-toxic nature, cost effectiveness, chemical stability and inertness, eco-friendliness, potential photocatalytic efficiency, *etc.*^13,14^. Even though both are effectively used, ZnO NPs are generally a better candidate for photocatalytic degradation of pollutants because of their high stability, non-toxicity,^15^ single oxidation state, natural abundance, suitable band gap^16^ and better biocompatibility.^17^ ZnO is a wide bandgap (3.37 eV)^18^ semiconductor which acts as an excellent photocatalyst for the decomposition of water pollutants.^19^ One of the top most priorities of the present-day research is for clean water in order to retain the ecosystem. Rapid growth of textile industries is one of the major causes for the water contamination. Dyes and its effluents from textile, food, and printing industries are the major pollutant released in water bodies. Generally, dyes are highly toxic and non-biodegradable because of its complex nature, causing serious threat to the ecosystem. methylene blue (MB) is a widely used dye in textile industries, which is extremely carcinogenic and toxic.^20^ Presently, more than 800 000 tonnes of dye are produced every year and its waste contents are released into water bodies by the industries.^21^ This is undoubtedly an alarming situation which needs to be addressed urgently. Various conventional methods such as coagulation, adsorption, ultrafiltration, ozonisation *etc.* are available, but they deal with the removal of dyes, effective in destroying some of the dyes only and need post treatment of secondary waste materials.^22^ So, semiconductor photocatalysis^23^ involving advanced oxidation process (AOP) has gained immense attention due to its effectiveness in degradation of the toxic dyes without generating any harmful products. Dye degradation using semiconductor photocatalyst is more environmentally friendly method, which can be a possible contender in waste water treatment.

The major worldwide challenge is to synthesise these metal oxide NPs in a cost effective, eco-friendly and sustainable manner. There are various conventional methods available such as physical methods (ball milling, solid state, physical vapor deposition *etc.*), chemical methods (sol–gel, coprecipitation, solution combustion, hydrothermal *etc.*) *etc* for synthesising metal oxide NPs.^1–12^ But these methods fail to address the problems related to toxicity, cost, waste production and environmental contamination. In order to overcome them, today's researchers have been immensely focusing on the green chemistry route for synthesizing metal oxides. Green approach towards the synthesis of metal oxide NPs is more eco-friendly,^24^ economically viable, socially accepted, safer, sustainable to environment *etc.* and account to increase in the yields of production.^25^ Synthesis of metal oxide NPs through greener route involves various biological components such as bacteria, fungi, algae, leaves, root *etc.* Among the available greener methods, the use of leaf extracts is the best way as it involves simplest, less investment, shorter time period, highly efficient, non-pathogenic^26^ and feasible process to prepare metal oxide at larger scale relative to bacteria, fungi and algae mediated synthesis. Various leaf extract such as *Cayratia pedata*^27^*Raphanus sativus* var. *Longipinnatus*^28^, *Tectona grandis* (*L.*)^29^, *Cassia fstula, Melia azedarach*^30^*etc.* were used as the stabilising agent for the synthesis of ZnO NPs. Therefore, in this regard, we have focused on synthesis of ZnO NPs from the *Lepidagathis ananthapuramensis* (LA) leaf extract, which is a highly effective green approach.

LA is a new species recently discovered in the Ananthapuram region of Kasaragod district, Kerala.^31^ It belongs to the *Acanthaceae* family, whose advanced studies related to medicinal or any other applications need to be done. The use of this LA leaf is a green mediated route for the synthesis of ZnO NPs. The leaf extract plays a crucial role in the synthesis process as it contains phytochemicals. These phytochemicals act as effective capping, stabilizing and reducing agents in the synthesis process. The phytochemicals present in the LA leaf extract are shown in the supplementary file,† which has been evaluated based on the method described in.^32,33^ The phytochemicals present in the LA leaf extract like alkaloids, flavonoids, terpenoids, glycosides, saponins, tannins, cardiac glycosides, reducing sugars, quinones, phenols *etc.* facilitate the production of ZnO NPs to give a high yield. In this one pot synthesis of ZnO NPs, the functional groups present in these phytochemicals play a binary role *i.e.*, they act as effective reducing and capping agents. The use of LA extract for the synthesis of ZnO NPs is a novel approach which can provide diverse nature of particles with the effectiveness in controlling its size and shape. Moreover, this leaf extract can provide better results related to other properties of ZnO NPs which has been clearly discussed in the entire work. Also, particles and crystallites size, shape, surface area and band gap are highly affected by the type of precursor, concentration of precursor, calcinations temperature, doping *etc.*^34,35^

Focusing on the eco-friendly approach, the present study emphasised on the novel LA leaf mediated synthesis of ZnO NPs and its capability of degrading the MB dye using solar driven photocatalytic process. The great band gap tuning, varied morphologies, outstanding stability, *etc.* of ZnO NPs enable them to achieve good photocatalytic outcomes. A systematic study of evaluating the photocatalytic efficiency of ZnO NPs was done based on various physicochemical parameters like importance of sunlight and ZnO catalyst, catalyst loading, concentrations of dye, zinc nitrate hexahydrate Zn(NO_3_)_2_·6H_2_O and LA extract, calcination temperatures and calcination hours. This systematic study can help to decide the best material for the excellent decomposition of water pollutant.

## 2. Experimental set-up

### 2.1 Materials

LA leaves were procured from Ananthapuram region (Anandashram), Kasaragod district, Kerala. Zn(NO_3_)_2_·6H_2_O (99% purity) was purchased from Sisco Research Laboratory (SRL) Pvt. Ltd. Methylene blue was purchased from Sigma-Aldrich. Distilled water was utilised for the synthesis.

### 2.2 Synthesis of LA leaf extract

LA leaves were collected, washed with distilled water, dried under shade for 1 week and hot air oven dried for couple of days. Then the leaves were grinded to fine powder using mixing grinder. This fine powder was used as a stock powder for future prospects.

10 g of fine powder of LA leaves were added to 250 mL distilled water taken in a 500 mL conical flask. The LA solution was stirred continuously for 2 hours at 80 °C. The solution was then cooled down to room temperature and filtered using a cotton cloth. The filtrate was used as the LA extract for the synthesis of ZnO NPs and stored in the refrigerator for future uses.

### 2.3 Synthesis of ZnO NPs using LA extract

Firstly, the synthesis of ZnO was done using 25 mL 0.1 M Zn(NO_3_)_2_·6H_2_O and 5 mL LA extract. Both the solutions were mixed well and stirred continuously at 60 °C for 4 hours in a 100 mL conical flask using a hot magnetic stirrer. As the reaction proceeds, the colour changes from green to dark green. The final product obtained was oven dried and calcined at 500 °C for 2 hours. The product calcined at 500 °C was grinded well using a mortar and pestle. Then this calcined sample was used for the characterisation to confirm the formation of ZnO NPs.

**Fig. 1:**
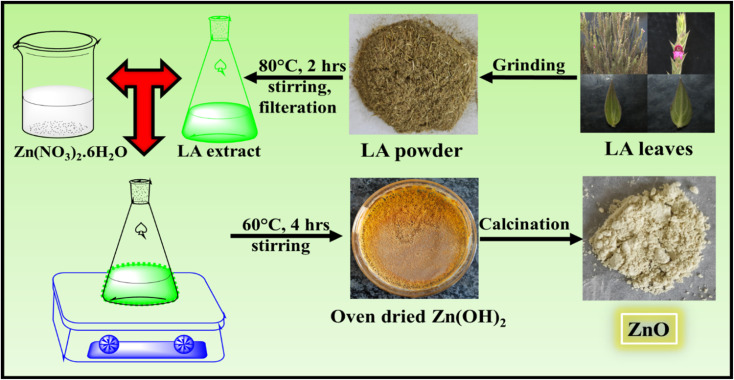
Schematic representation of synthesis of ZnO NPs using LA leaf template.

Further property study of ZnO NPs was done with respect to concentration of Zn(NO_3_)_2_·6H_2_O, concentrations of LA extract, calcination temperatures and calcination hours following the same procedure mentioned earlier. (Table 1) The following table shows the different parameter used for the property studies and their corresponding sample name which is mentioned in their characterisation plots (Fig. 1).

**Table 1:** Synthesis of ZnO NPs based on variable parameters

Variable parameters	No. of samples synthesised	Sample name	Concentration of Zn(NO_3_)_2_	Leaf extract	Calcination temperature	Calcination hours
Concentration of Zn(NO_3_)_2_	1	ZnO-0.025	25 mL 0.025M	5 mL	500 °C	2
	2	ZnO-0.05	25 mL 0.05 M			
	3	ZnO-0.10	25 mL 0.10 M			
	4	ZnO-0.15	25 mL 0.15 M			
	5	ZnO-0.20	25 mL 0.20 M			
	6	ZnO-0.30	25 mL 0.30 M			
	7	ZnO-0.50	25 mL 0.50 M			
	8	ZnO-0.70	25 mL 0.70 M			
	9	ZnO-1.00	25 mL 1.00 M			
Concentration of LA extract	10	ZnO-1 : 5	25 mL 0.10 M	5 mL	500 °C	2
	11	ZnO-2 : 5		10 mL		
	12	ZnO-3 : 5		15 mL		
	13	ZnO-4 : 5		20 mL		
	14	ZnO-5 : 5		25 mL		
Calcination temperature	15	ZnO-500	25 mL 0.10 M	5 mL	500 °C	2
	16	ZnO-600			600 °C	
	17	ZnO-700			700 °C	
	18	ZnO-800			800 °C	
	19	ZnO-900			900 °C	
	20	ZnO-1000			1000 °C	
Calcination hours	21	ZnO-2	25 mL 0.10 M	5 mL	500 °C	2
	22	ZnO-4				4
	23	ZnO-6				6

The crystallite size and crystallinity percentage are calculated using the following equations:
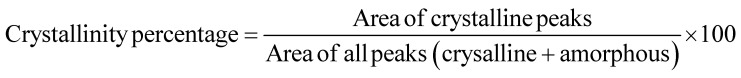
Crysatallite size, *D* = 0.9*λ*/*β* cos *θ*where *λ* is the wavelength of X-ray used *i.e.*, 0.154 nm, *β* is the full width at half maximum, *θ* is the Bragg's angle. All the parameters' units are used in radian and the obtained crystallite size is calculated in radian only.

### 2.4 Photocatalytic set-up

The photocatalytic studies were done using ZnO NPs as a semiconductor photocatalyst and MB dye as the model dye. The photocatalytic activity of ZnO NPs was analysed based on the observation of degradation (decolouration) of MB dye. The photocatalytic efficiency is calculated using the following equations; [*A*_0_ − *A*/*A*_0_] × 100 or [*C*_0_ − *C*/*C*_0_] × 100 where *A*_0_ and *A* is the initial (0 minute) and final (90 minute) absorbance values of the MB solution without and with ZnO semiconductor photocatalyst and *C*_0_ and *C* are the 0 minute and 90 minute MB dye concentrations without and with ZnO semiconductor photocatalyst at 0 minute and 90 minutes respectively.

The photocatalytic experimental set-up is developed by exposing reaction mixture (ZnO-MB dye solution) in sunlight under constant stirring of 500 rpm in a magnetic stirrer. The solar light intensity was measured with the help of lux meter. Initially for trial basis, 5 mg of ZnO-1 : 5 was used and added to the 50 mL of 10^−5^ M MB dye solution. The solution then stirred continuously under sunlight. At a regular interval of 15 minutes, 5 mL of reaction mixture is taken out using a micropipette by switching of the stirring process. Up to 90 minutes, 6 such aliquots were taken and each mixture was centrifuged at 10 000 rpm for 10 minutes. Each mixture was then analysed using the UV-visible spectroscopy over the wavelength range of 200–800 nm, in order to check the absorbance values. The change in absorbance values is the guidance towards the MB degradation studies.

The similar procedure was followed for the systematic study of photocatalysis based on various parameters *i.e.*, influence of sunlight and no sunlight (dark reactions), catalyst loading, effect of concentrations of Zn(NO_3_)_2_·6H_2_O precursor, LA extract and MB dye, effect of calcinations temperatures and effect of calcination hours. Most of the photocatalytic activity of ZnO NPs based on various parameters has been compared with ZnO-LA and 5 mg ZnO photocatalyst is used for the photocatalytic studies.

### 2.5 Characterisation techniques

The structural characterisation was done based on XRD analysis with the help of PAN analytical X'Pert Pro diffractometer using the wavelength of Cu-Kα (*λ* = 1.54060 Å) radiation. The materials were slow scanned in the range of 20–80° with the step size of 0.02. The optical properties were studied with the help of UV-1800 Shimadzu spectrophotometer. The characteristic structural information of ZnO was analysed using LabRam HR evolution Raman spectrometer having a 633 nm laser (Horiba scientific, France). FT-IR spectroscopy was performed using PerkinElmer 100 spectrometer (USA) in the scan range of 4000–400 cm^−1^. The electronic states were analysed using Thermo Scientific K-Alpha XPS instrument having monochromatic Al Kα radiation, *E*_photon_ = 1486.6 eV. TGA was employed to check the chemical and thermal stability using TA Q500 Instrument (USA) in the range of 0–800 °C at 5° min^−1^ heating rate under nitrogen atmosphere. The BET was employed to check the surface area using Microtrac BEL instrument (BELPREP vacII, JAPAN). The surface and elemental composition were studied using FE-SEM/EDAX analysis with the help of Hitachi-S520 (Oxford link ISISSEM Model), Japan.

## 3. Results and discussions

### 3.1 Possible mechanism of synthesising ZnO NPs from LA leaf template

**Fig. 2:**
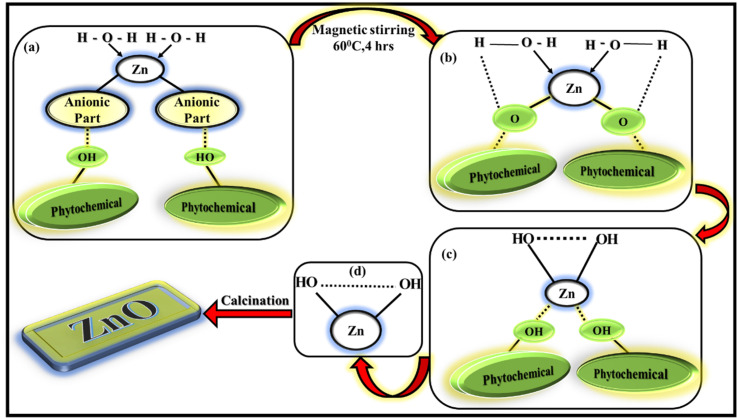
Possible mechanism of formation of ZnO NPs.

The importance of leaf extract for synthesising ZnO NPs is because of the availability of phytochemicals present in leaf extract. The phytochemicals contain different types of functional groups like ketone, aldehyde, hydroxyl, carboxylic acid *etc.* which helps in the formation of ZnO NPs involving chemical reduction and oxidation process. The present LA extract contain alkaloids, flavonoids, phenols, quinones *etc*, as mentioned in ESI† file. These phytochemicals will act as effective capping agents as well as reducing-oxidising agents in the synthesis process. The exact mechanism of forming ZnO NPs from leaf extract is not yet understood completely, several research works are being performed to study the exact mechanism. (Fig. 2) The possible mechanism of forming ZnO NPs *via* greener way involves two steps as mentioned in:^35^

1. Formation of Zn(OH)_2_ from the corresponding Zn(NO_3_)_2_·6H_2_O salt.

2. Calcination of the subsequent reaction product into ZnO NPs.

Zn^2+^ ions present in the zinc electrolytic solution [Zn(NO_3_)_2_·6H_2_O solution]is reduced to the stabilised compound by chelating to the phytochemicals such as flavonoids and polyphenols, present in the leaf extract during the reaction. The anionic part of zinc salt will be eliminated as corresponding acid by taking proton from the phytochemicals. The water molecules will try to donate its proton to the corresponding phytochemicals. As a result, the hydroxyl group present in the reaction mixture (biomolecule or in solution) will try to make bond with stabilised metal species leading to the generation of zinc hydroxide (Zn(OH)_2_). All these processes occur simultaneously. The obtained solution is oven-dried overnight and a yellow–orange pre-product was obtained which was calcined to eliminate the hydroxides and the impurities to finally obtain ZnO NPs.

### 3.2 Analysis of synthesized ZnO NPs

#### 3.2.1 XRD analysis

**Fig. 3:**
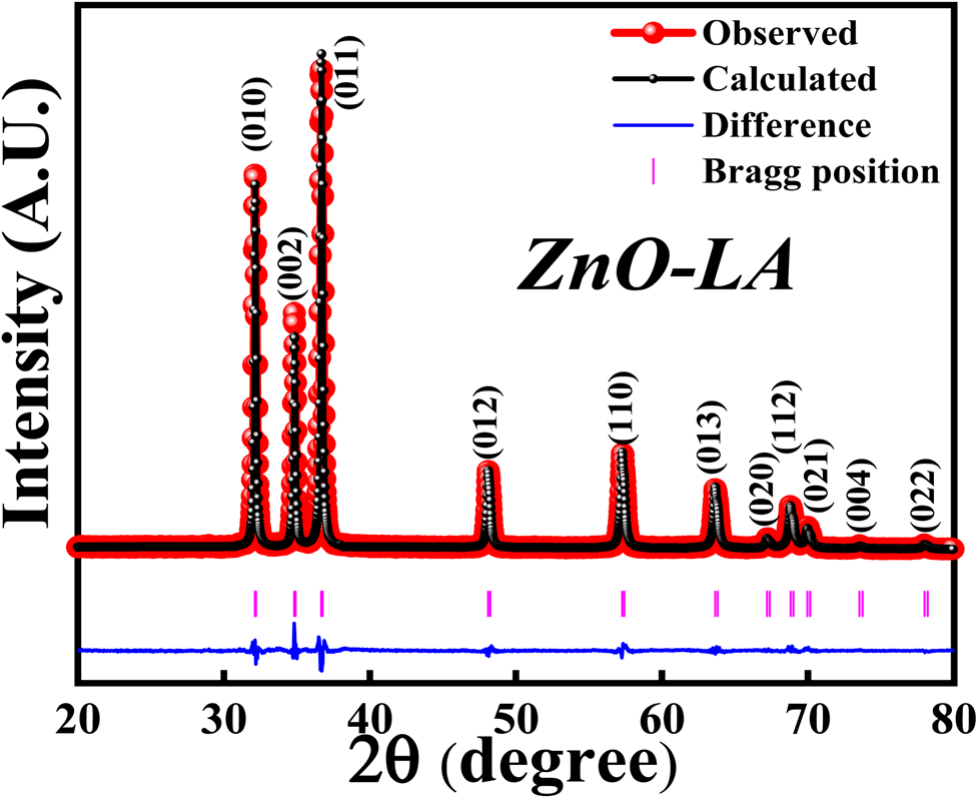
XRD-Rietveld refinement of ZnO NPs.

The XRD analysis was employed to know the structural formation and phase purity of ZnO NPs as well as to know about the crystallinity and crystallite size. The sample was analysed over the scan angle from 20° to 80° with the step size of 0.02. The obtained XRD data was refined using the Full Prof software. Fig. 3 represents the Rietveld refinement of ZnO and the refined parameters are shown in Table 2. The sharp intense peaks of synthesised ZnO corresponds to (010), (002), (011), (012), (110), (013), (020), (112), (021), (004) and (022) lattice planes diffracted at 31.76°, 34.43°, 36.26°, 47.55°, 56.59°, 62.87°, 66.38°, 67.95°, 69.09°, 72.85° and 76.96°. These crystalline peaks are in well accordance with the single-phase hexagonal wurtzite crystalline structure of ZnO NPs with the space group *P*6_3_*mc*, as per Inorganic Crystal Structure Database (ICSD ref. Code: 98-008-1255). The absence of extra peaks indicates the high purity nature of synthesised ZnO NPs. The percentage of crystallinity and crystallite size were calculated using the peak area and Debye–Scherrer's equation:
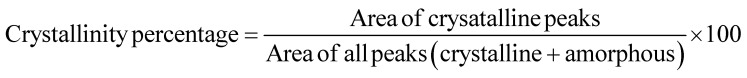
Crysatallite size, *D* = 0.9*λ*/*β* cos *θ*

**Table 2:** Rietveld refinement parameter obtained for ZnO NPs

Sample	ZnO
**Cell parameter**	
Crystal structure	Hexagonal
Space group	*P*6_3_*mc*
Point group	6 mm or C_6v_
*a* (Å)	3.25
*b* (Å)	3.25
*c* (Å)	5.21
*α*	90
*β*	90
*ϒ*	120
Volume (Å^3^)	47.61
Density (g cm^−3^)	5.68

**Positional parameter**	
Zn *x*	0.3333
Zn *y*	0.6667
Zn *z*	0.0024
O *x*	0.3333
O *y*	0.6667
O *z*	0.3814

**Site occupation**	
Zn	1.0
O	1.0

**Agreement factors**	
*R*_WP_	15.8
*R*_P_	11.7
*R*_EXP_	12.0
_*χ*_^2^	1.74

It was observed that the synthesized ZnO NPs have the 81.40% crystallinity with the average crystallite size of 28.12 nm. This indicates that the sample is highly crystalline possessing nano-crystallites. Further study of XRD analysis with respect to effect of concentrations of precursor and LA extract, effect of calcinations temperatures and calcinations hours are discussed later.

#### 3.2.2 UV-visible analysis

**Fig. 4:**
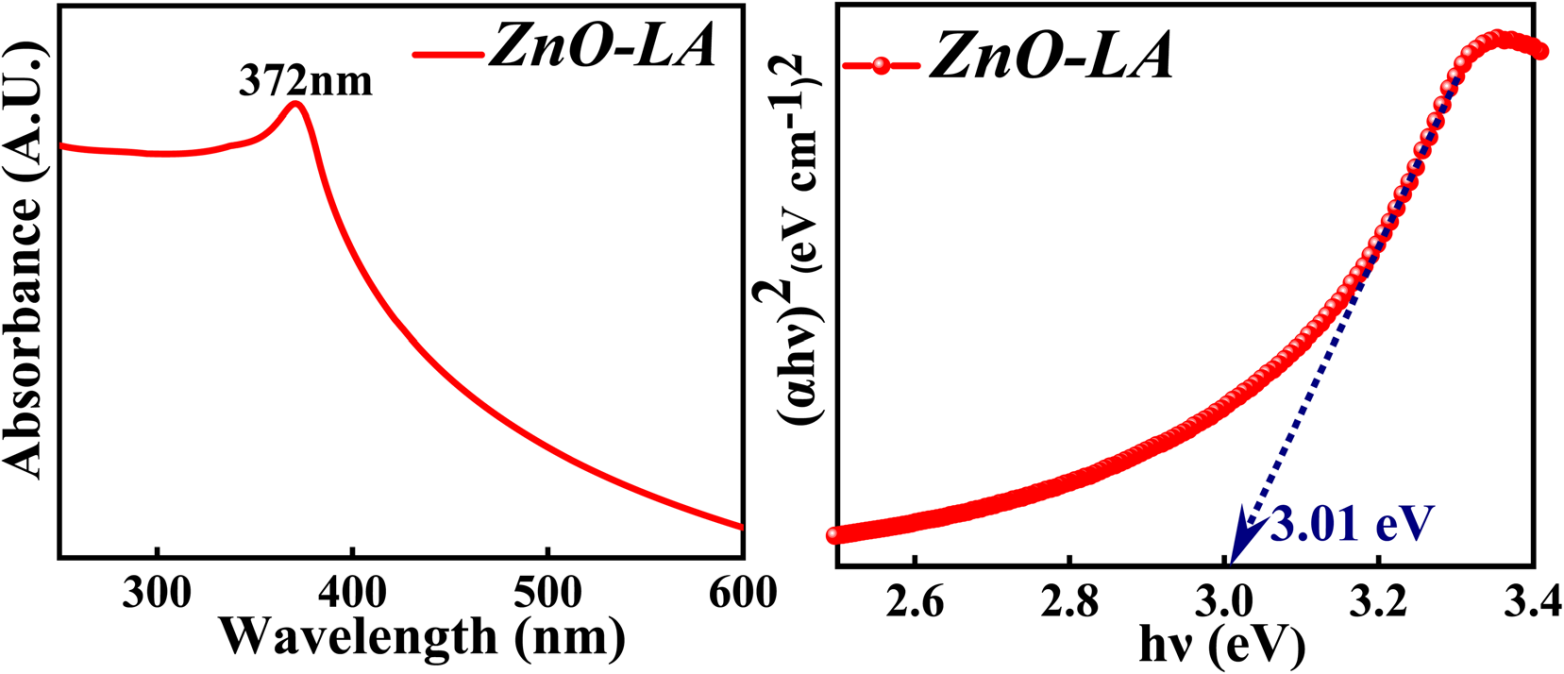
(Left) UV-visible spectrum and (Right) Tauc plot of ZnO NPs.

The UV-visible spectroscopy was employed to know the absorption property and thereby evaluate the band gap of ZnO NPs. The sample was analysed in the range 200–800 nm and the band gap is calculated by the Tauc method. The obtained absorption spectrum is shown in Fig. 4 along with the Tauc plot. The maximum absorbance is observed at 372 nm which can be due to the intrinsic semiconductor band gap transition having the surface plasmon resonance as well as quantum size effect phenomena. The characteristic narrow peak obtained at 372 nm is in agreement with the earlier reports.^36,37^ The optical band gap obtained for ZnO NPs is around 3.01 eV which is slightly lower than the previously reported or standard band gap of ZnO (3.10 eV–3.37 eV).^38,39^ Generally, the band gap reduces with respect to particle size, as the particle size increases ultimately the band gap decreases as a consequence of quantum size effect. But it is not always true that the band gap depends only on particle size factor. The decrement in the band gap of ZnO NPs can also be attributed to various factors as well, such as synthesis process, morphology,^40^ lattice strain, grain size, defect and roughness of the surface.^41^ It is difficult to judge the actual factors that has changed the optical gap, but it can be either one or combination of former factors mentioned. Moreover, the appearance of sharp intense peak along with no other small peaks in the absorption spectrum indicates highly pure nature of synthesised ZnO NPs.

#### 3.2.3 Raman analysis

**Fig. 5:**
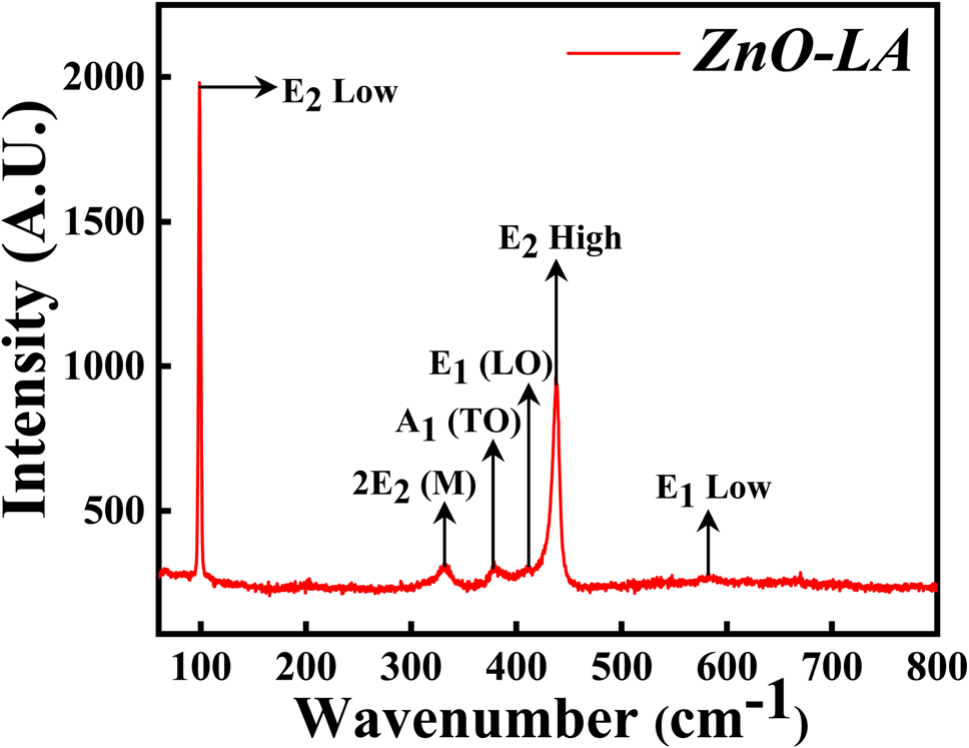
Raman spectrum of ZnO NPs.

The Raman spectroscopy was performed in order to get the confirmation of characteristic hexagonal crystalline structure of ZnO NPs. The sample was analysed over the range of 0–800 cm^−1^ which is the finger print region of ZnO NPs. The Raman spectrum depicted in Fig. 5 shows the characteristic peaks of hexagonal wurtzite structure of ZnO NPs which corresponds to C_6v_ point group whose irreducible representation is expressed as A_1_ + 2B_1_ + E_1_ + 2E_2_. Among this, polar modes A_1_ and E_1_ are Raman and infrared active whereas E_2_ is also a polar mode which is Raman active but infrared inactive and B_1_ mode is both Raman and infrared inactive.^42^ Moreover, A_1_ and E_1_ modes are divided into two modes *i.e.*, transverse optical (TO) phonons and longitudinal optical (LO) phonons. E_2_ consists of low frequency mode (E_2_ low) and high frequency mode (E_2_ high). The intense peak at 99.3 cm^−1^ belongs to E_2_ low phonon mode which is due to the vibrations of Zn lattice and the dominant peak at 438.1 cm^−1^ belongs to E_2_ high frequency mode which is because of the lattice oxygen vibrations. The small broad peak at 332 cm^−1^ corresponds to multi-phonon mode (E_2M_). This broad peak is also known as second-order mode caused by the multi-phonon process (31) (combined vibrations associated with the ZnO lattice). The small intense peaks at 378.1 cm^−1^, 412 cm^−1^ and 583.5 cm^−1^ are attributed to A_1_(TO), E_1_(LO) and E_1_ low phonon modes. Generally, these peaks arise due to the structural defect present in the ZnO lattice (31, 29). More the deficiency, more the peaks are affected (especially E_1_(LO) being affected strongly^43^) causing a change in the peak intensity. Sometimes, peaks are also generated at 275, 510 and 643 cm^−1^ which are extra phonon modes arise due to the defects.^44,45^ In the present case, the intensity of these peaks are very small suggesting that synthesised ZnO NPs are having negligible defects. These results suggest that ZnO NPs are of high purity nature having fewer defects.

#### 3.2.4 XPS analysis

**Fig. 6:**
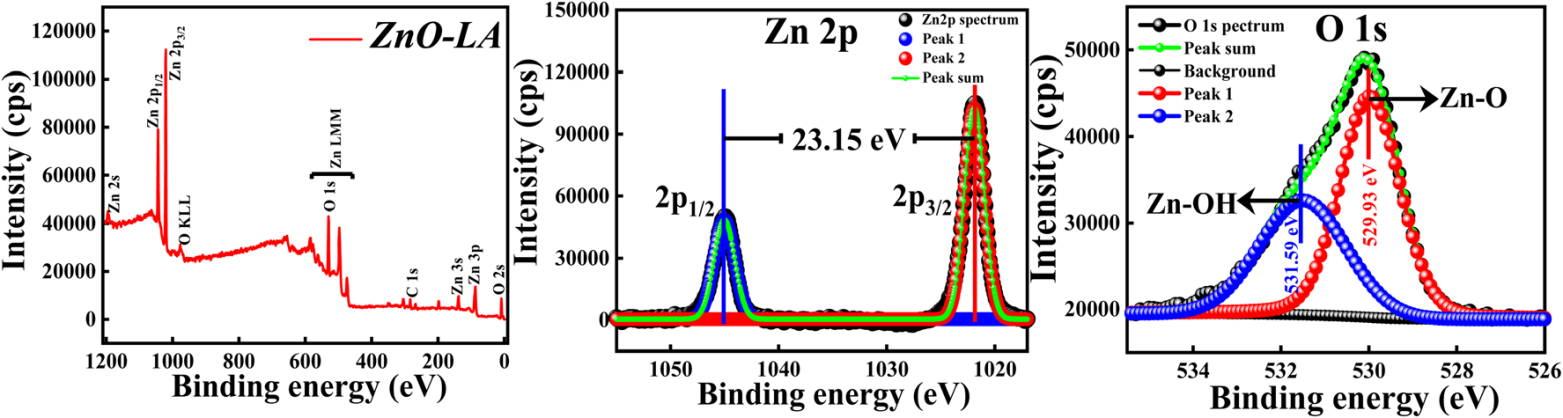
(Left) XPS survey spectrum; (Middle) XPS high-resolution slow scan of Zn 2p spectrum and (Right) O 1s spectrum of ZnO NPs.

The XPS analysis of ZnO NPs was carried out to explore the elemental compositions, defects and oxidation states of Zn element present in the material. All the binding energies (B.E.) measured were corrected with respect to C 1s peak positioned at 284.6 eV. The wide scan spectrum manifested in Fig. 6 clearly ensured that no foreign materials are present on the surface of the nanomaterials. The high-resolution scan of Zn 2p and O 1s spectra are depicted in the Fig. 6 along with the deconvolution. The slow scan of Zn 2p spectrum shows two characteristic strong peaks located at 1021.9 eV and 1045.05 eV, which belong to the binding energies of Zn 2p_3/2_ and Zn 2p_1/2_ respectively. The difference between the B.E. of Zn 2p_3/2_ and Zn 2p_1/2_ peaks is 23.15 eV, which is a characteristic spin–orbit splitting binding energy of ZnO. The deconvolution of Zn 2p spectrum shows that both the peaks are fitted with one peak only. This suggests that Zn existed as Zn^2+^ ions instead of metallic Zn particularly. Similarly, the slow scan of O 1s spectrum is deconvoluted into two subpeaks positioned at 529.93 eV and 531.59 eV, which are attributed to the lattice oxygen (Zn–O) and oxygen of hydroxyl group adsorbed on ZnO NPs *i.e.*, moisture adsorption (Zn–OH). If the deconvolution of O 1s spectrum has resulted in 3 sub peaks, surface defects will be present. This result clearly suggests that chance of defect generation in the sample may be less since the O 1s spectrum is fitted with two peaks only. All these results are in well agreement with the previous reports.^46^ The main conclusion from the XPS analysis is that synthesised ZnO NPs are free from impurities involving fewer defects.

#### 3.2.5 FT-IR analysis

**Fig. 7:**
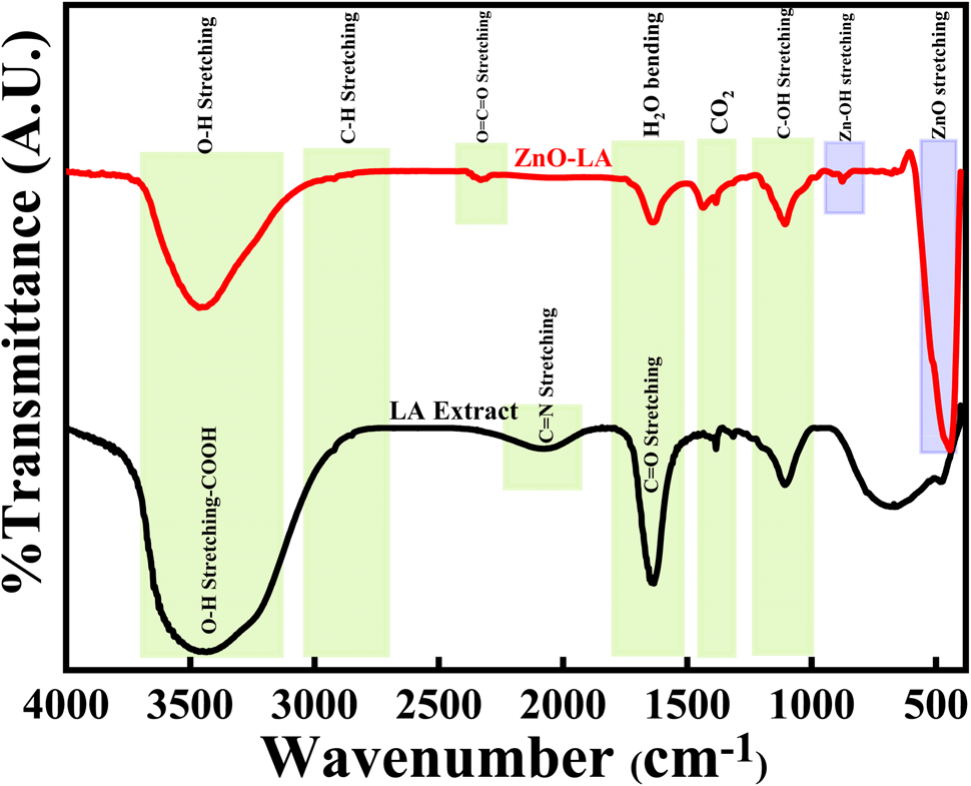
FT-IR spectrum of ZnO NPs and LA leaf extract.

The FT-IR analysis of ZnO NPs were done along with LA leaf extract over the infrared range of 400–4000 cm^−1^ using KBr pellet, as represented in Fig. 7. The LA leaf extract showed peaks around 3690–3050 cm^−1^(very broad), 2070 cm^−1^ (broad), 1645 cm^−1^ (sharp intense) and 1105 cm^−1^ (medium strong) which correspond to OH stretching frequency of carboxylic acid, C=N stretching frequency, C=O/C=C stretching frequency and C–OH stretching frequency respectively. These peaks arised due to various phytochemicals content such as quinones, alkaloids *etc.* present in the leaf extract. However, some of the peaks were absent in the case of calcined ZnO NPs. ZnO NPs showed characteristic strong intense peak around 445 cm^−1^, small intense shoulder peak around 525 cm^−1^ and small intense at 874 cm^−1^ which belong to Zn–O and Zn–OH stretching vibrations. All these vibrations are due to the Zn–O bond present in the crystal lattice. Also, ZnO NPs showed peaks at 3640–3140 cm^−1^, 1632 cm^−1^ and 1420 cm^−1^ that are attributed to the O–H stretching, H_2_O bending vibrations of adsorbed moisture and atmospheric CO_2_ interference during analysis. All the peaks obtained are well matched with earlier results^47–51^

#### 3.2.6 TGA analysis

**Fig. 8:**
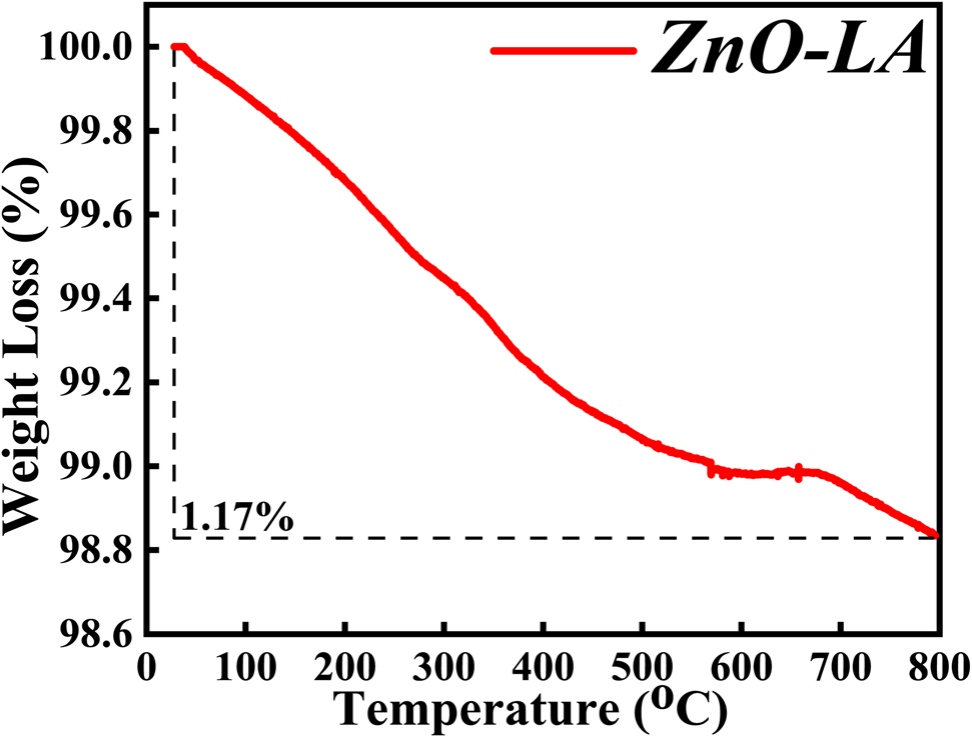
Temperature variable thermal study of ZnO NPs.

The TGA characterisation was carried out in order to determine the thermal and chemical stability of synthesised ZnO NPs. The sample was analysed after one year of preparation in the range of 30 °C to 800 °C at a ramp rate of 5 °C min^−1^. The obtained thermal plot of ZnO NPs is shown in Fig. 8. It is observed that the overall percentage of degradation of the ZnO NPs is 1.17% which can be neglected. The overall 1.17% decomposition of ZnO NPs may be due to the elimination of adsorbed moisture from the nanoparticles surface and removal of some impurities. The main observation from the TGA analysis is that the material is highly stable for prolonged duration.

#### 3.2.7 BET analysis

**Fig. 9:**
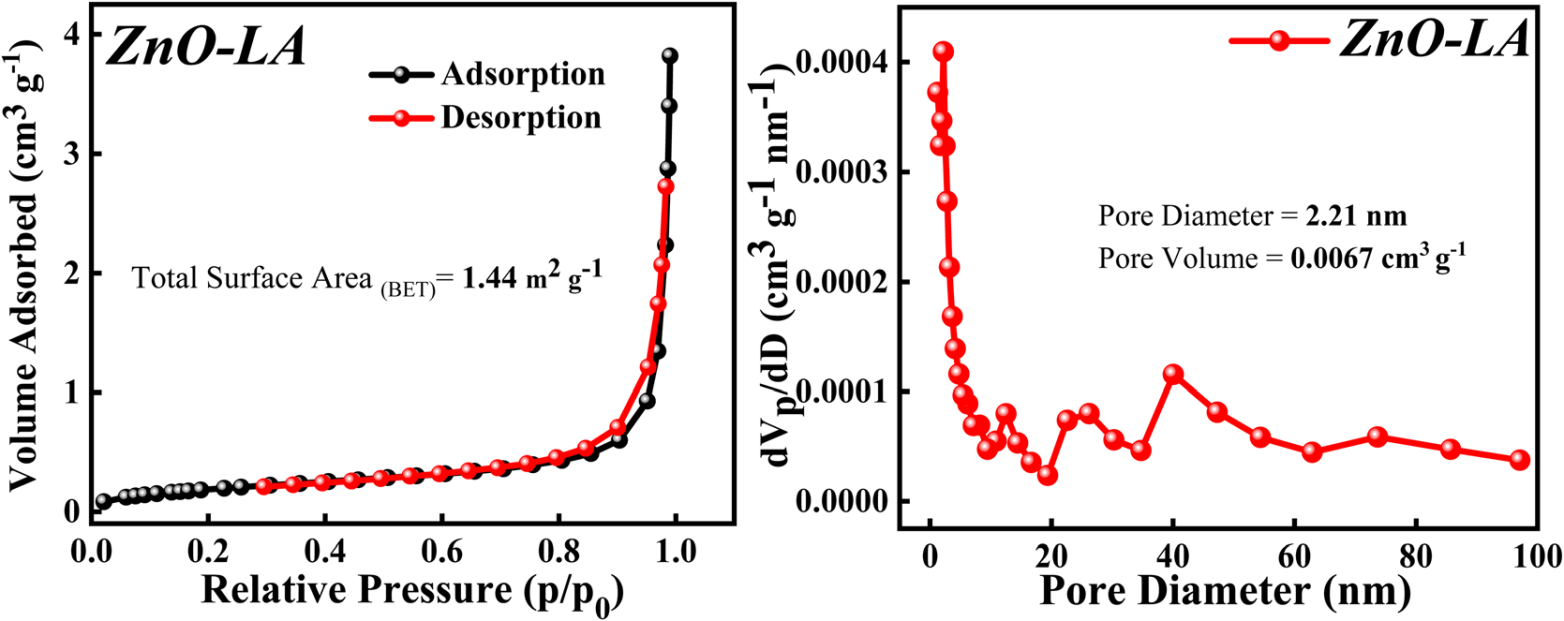
(Left) N_2_ adsorption–desorption isotherm, (Right) BJH plot of ZnO NPs.

The BET analysis was performed to know about the surface area, pore diameter and pore volume of the sample. The N_2_ adsorption–desorption isotherm along with Barrett–Joyner–Halenda (BJH) plot are depicted in Fig. 9. The N_2_ adsorption–desorption isotherm shows that the isotherm is of type 4 involving H_3_ hysteresis loop noticed in the *p*/*p*_0_(relative pressure) range of 0.84–0.98. This result indicates that the ZnO NPs is a mesoporous material showing slit shaped pores.^52^ The total surface area obtained using BET method is 1.44 m^2^ g^−1^. This extremely low surface area can be due to the agglomeration of particle and the measured pore size and pore volume is 2.21 nm and 0.0067 cm^3^ g^−1^ respectively, which signifies ZnO NPs as a mesoporous material.

#### 3.2.8 FE-SEM & EDAX analysis

**Fig. 10:**
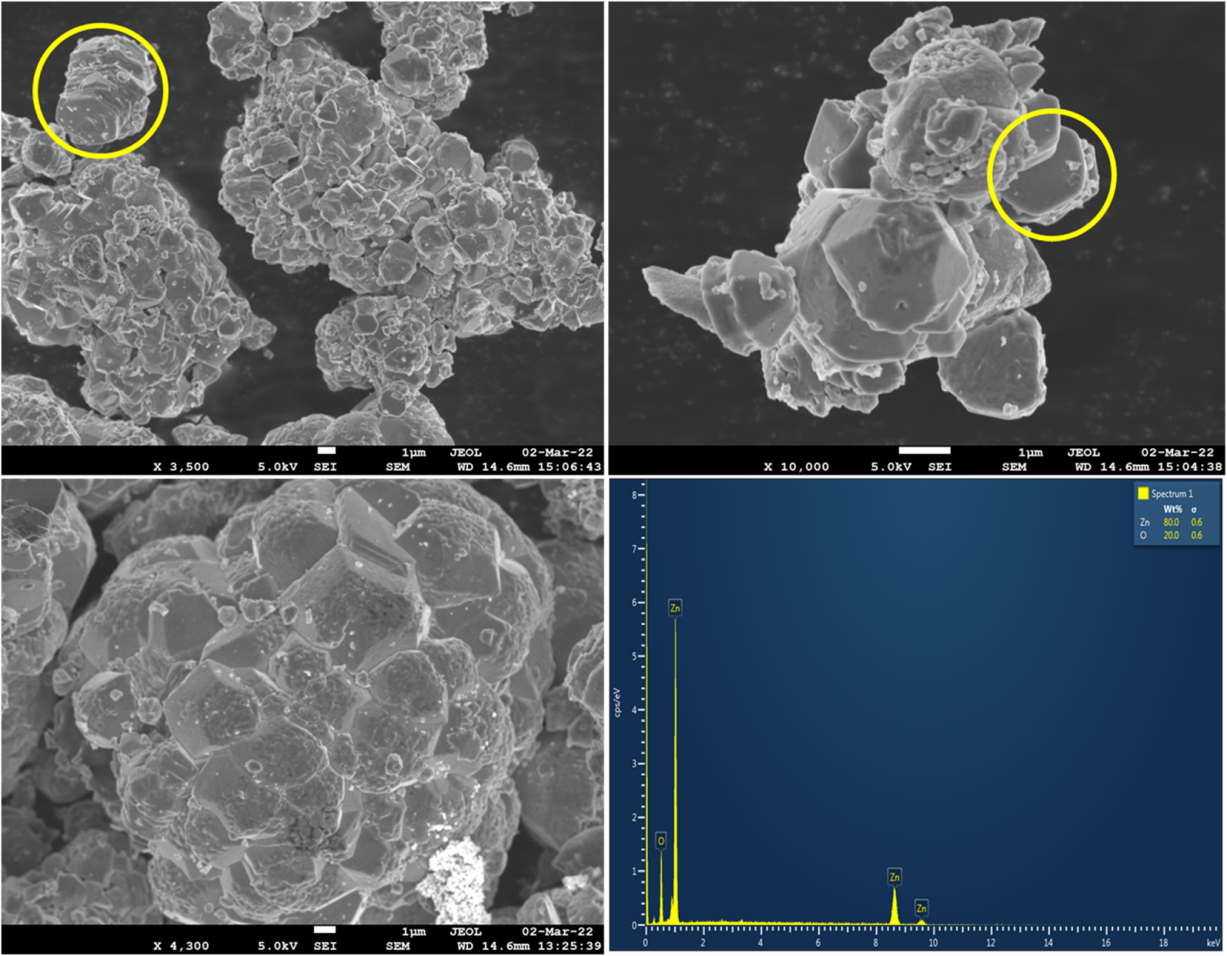
FE-SEM micro images of ZnO and EDAX spectrum of ZnO NPs.

The surface morphology of ZnO NPs was taken out using FE-SEM analysis and the elemental analysis was carried out using EDAX technique. The obtained FE-SEM micro images are shown in the Fig. 10 along with the elemental spectrum of ZnO. The micro images of FE-SEM clearly showed that some of the ZnO particles are in hexagonal morphology (also depicted in SEM image-Fig. S1†), but most of the particles are agglomerated^27^ which resulted in the stacking morphology (marked with yellow circle). Sometimes, the agglomeration of the particles resulted in the stacking morphology looked like umbrella type (mushroom form), flower type, honey comb cage like morphologies. The micro-images also indicated that most of the particles are in micrometre range due to agglomeration. The existence of nanoparticles was confirmed from HRTEM analysis (Fig. S2†-histogram plot). Hence the synthesised ZnO NPs are mostly agglomerated showing stacking morphology.

The elemental analysis of ZnO NPs showed four absorption peaks pertaining to Zn and O with the weight percentage of 80 and 20. There are no additional peaks observed indicating that the synthesised ZnO NPs is of high purity.

#### 3.2.9 TEM and HR-TEM analysis

**Fig. 11:**
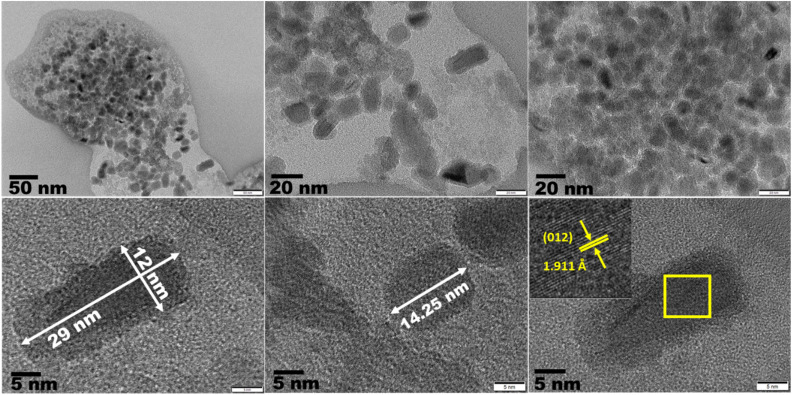
TEM images and lattice fringes patterns of ZnO NPs.

TEM and HR-TEM was employed to study the morphology and particle size of ZnO NPs and the selected area electron diffraction (SAED) was done to confirm the crystal planes of ZnO NPs. Fig. 11 displays the TEM images of ZnO NPs in different magnifications and Fig. S2† represent the histogram, SAED and lattice fringe pattern of ZnO NPs. It is clearly observed that ZnO NPs were formed with different morphologies like rod, hexagonal, and spherical shapes. The examination of crystal lattice planes, d-spacing, and particle size distribution were performed using Image J software. The average particle size of ZnO NPs was around 10–15 nm (displayed in histogram), which has been evaluated based on the selection of 100 NPs (mostly spherical type particles) from different TEM images. It is also noticed that the length and breadth of an individual rod type particle is around 29 nm and 12 nm respectively. Similarly, the particle size of an individual hexagonal shaped ZnO NPs (seen in the HR-TEM image) is 14.25 nm. So, it is clear that the formed ZnO NPs are of polydisperse nature (also confirmed from dynamic laser scattering (DLS) measurement mentioned in Fig. S3†). HR-TEM images clearly showed lattice fringes without any defects indicating high degree of crystallinity. The calculated interplanar *d*-spacing of neighbouring lattice fringes is approximately 1.89–1.91 Å, 1.473 Å and 1.305 Å for (012), (013) and (004) crystal planes. The SAED pattern clearly indicated the concentric rings which have been matched with the (010), (011), (012), (110), (004) and (023) crystal lattice planes of hexagonal wurtzite crystalline structure of ZnO NPs (ICSD ref. Code: 98-008-1255). Hence, the synthesised ZnO NPs are polydisperse in nature and have a high degree of crystallinity.

### 3.3 Crystallinity and crystallize size studies based on XRD analysis

#### 3.3.1 Effect of concentration of Zn(NO_3_)_2_·6H_2_O precursor

**Fig. 12:**
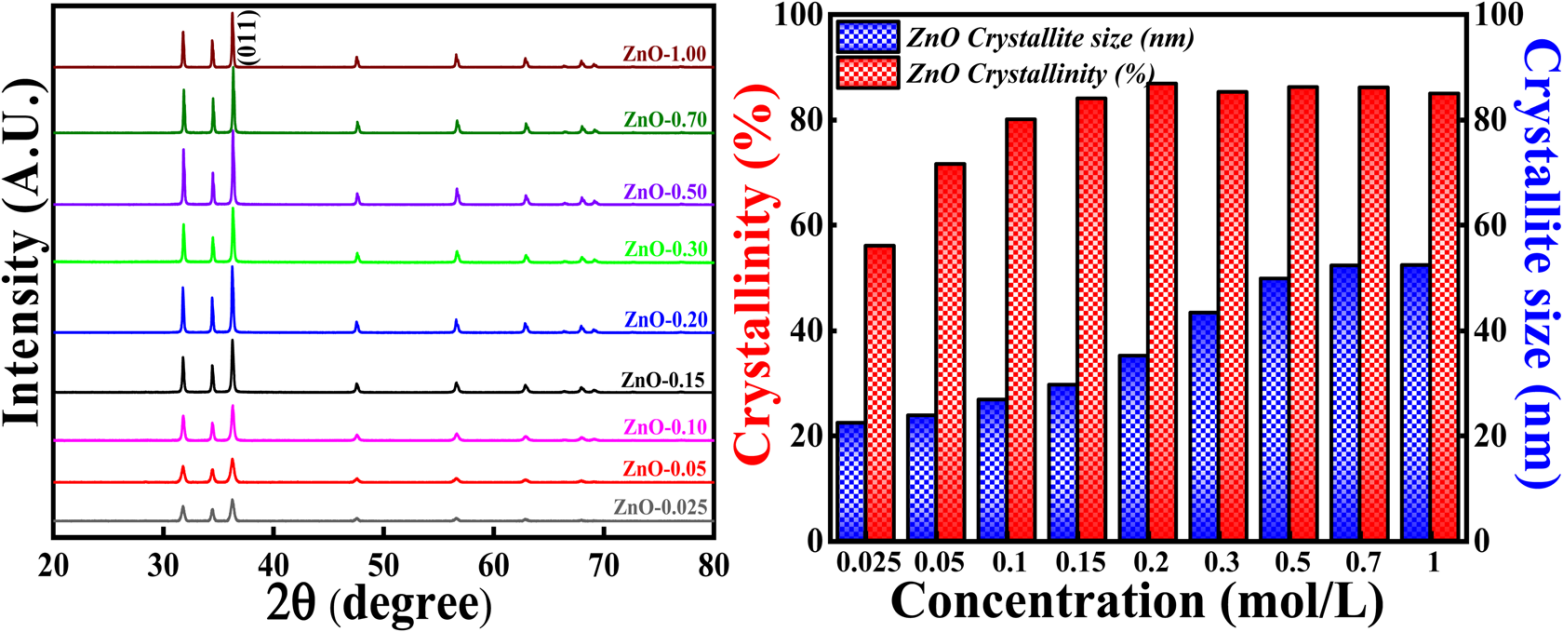
(Left) XRD patterns and crystallinity, (Right) crystallite size studies based on different molar concentration of Zn(NO_3_)_2_·6H_2_O.

The crystallinity and crystallite size studies were analysed and calculated based on varying the concentration of Zn(NO_3_)_2_·6H_2_O and the obtained XRD patterns are depicted in Fig. 12. All the XRD patterns synthesised using different concentrations of precursors showed single hexagonal phase of wurtzite crystalline structure which are in well agreement with ICSD ref. Code: 98-008-1255. There are no additional peaks indicating that there are no impurities or any secondary (mixed) phases as the concentrations are varied. It is noticed that the intensity of each diffraction plane is enhancing but the (011) peak is of great interest due to its intense and sharpest nature. Degree of crystallinity and crystallite size were calculated with respect to (011) plane. It is clearly observed that as the concentration of precursors is increased, the crystallinity percentage and crystallite size are also increasing up to a particular concentration. The crystallinity percentage increased to 87.10% for 0.2 M concentration, after that crystallinity percentage almost remains the same. Similarly, the crystallite size increased up to 52.3 nm for 0.7 M concentration. The increase in crystallinity percentage is reflected in the (011) plane as it became more intense and sharper as the concentration is increased. Moreover, from Fig. S4,† there are slight variations in Bragg's angle, FWHM and peak intensity for (011) plane as the concentration is varied. The increase in crystallite size with the increase in the precursor concentrations can be due to the agglomeration of crystallites as a result of higher concentration. Also, the rate of conversation of Zn(NO_3_)_2_·6H_2_O to Zn(OH)_2_ increases as the collision (reduction and oxidation process) of highly concentrated electrolytes with phytochemicals increases. Moreover, there are chances of defect generation (oxygen vacancies (O_v_), zinc interstitials (Zn_i_) *etc.*) as a result of agglomeration of crystallites. However, there is no direct relationship between crystallinity and crystallite size. Hence, the highest crystallinity and crystallite size is obtained for 0.2 M and 0.7 M, which means that long range order of atoms, is attained for 0.2 M with crystallite size around 35.12 nm. Therefore, in order to get good crystallinity and lowest crystallite size, concentration of precursors should be optimised. In the present study, 0.1 M Zn(NO_3_)_2_·6H_2_O is used as the optimised concentration for further study as it has 81.40% crystallinity with the crystallite size of 28.12 nm.

#### 3.3.2 Effect of concentration of LA extract

**Fig. 13:**
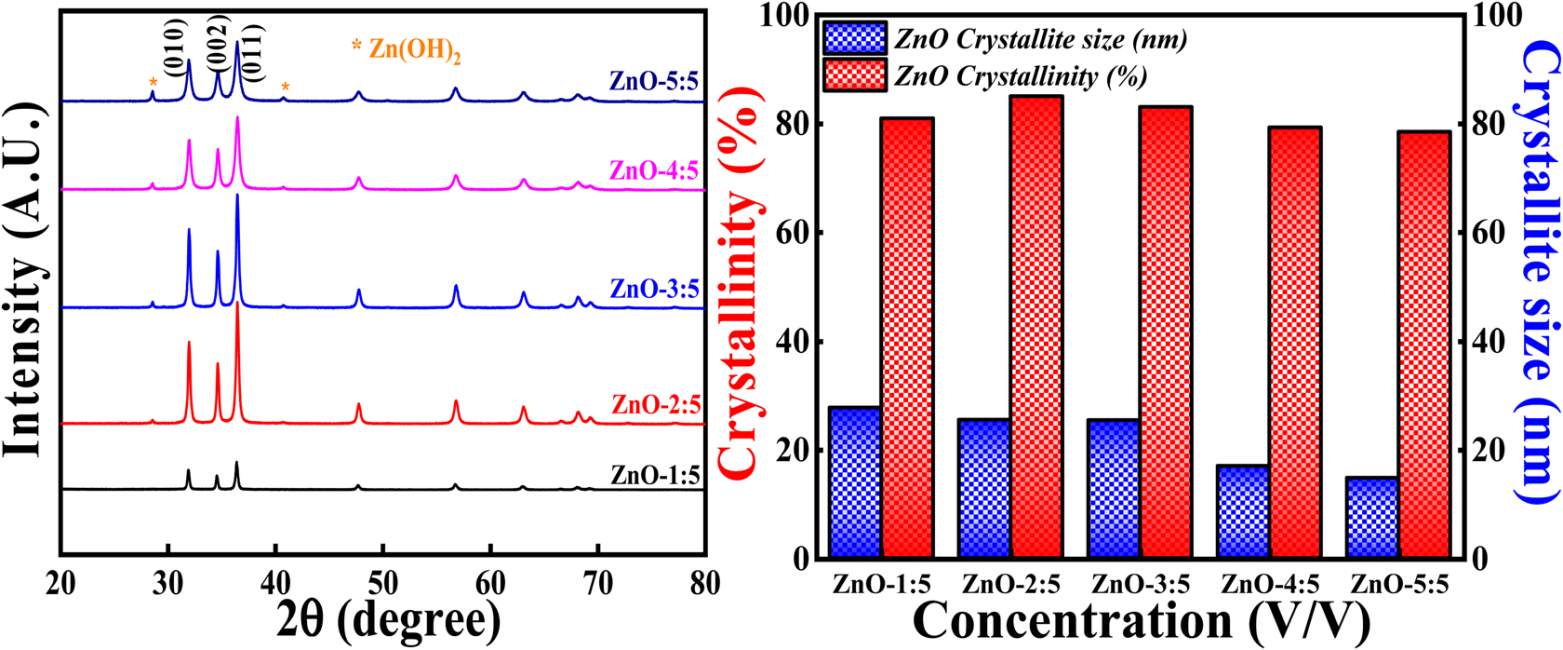
(Left) XRD patterns and crystallinity, (Right) crystallite size studies based on volume ratio LA: Zn(NO_3_)_2_·6H_2_O.

The effect of concentration of LA extract on the degree of crystallinity and crystallite size is explored by keeping the calcinations temperature, calcinations hours and concentration of precursor constant. The obtained XRD profiles are represented in Fig. 13 along with the bar graphs showing crystallinity percentage and crystallite size. It is interesting to say that XRD patterns showed generation of Zn(OH)_2_ impurity phase along with the pure ZnO as the volume of LA extract is increased. The generation of Zn(OH)_2_ impurity phase is increased with the increase in the LA content. The obtained impurity phase is in well agreement with earlier reported results.^53,54^ The obtained ZnO crystalline peaks are in well concordance with the ICSD ref. Code: 98-008-1255.

The broadness of the highly intense (011) peak can be seen as the concentration of LA is increased, as represented in Fig. 13 and S5.† The broadness of this peak signifies the formation of the small crystallites due to the large FWHM value (Scherrer's equation). This is clearly reflected in the crystallite size also, as the concentration of LA extract is increased, crystallite size simultaneously decreased. The lowest crystallite size is obtained for ZnO-5 : 5 *i.e.*, 14.72 nm. The decrement in the crystallite size may be due to the increase in the concentration of stabilising agent (effective capping), which will prevent the particles (crystallites) to agglomerate.^55^ So, the smaller crystallite size can be obtained for ZnO NPs involving more LA content in the synthesis process. But increase in the concentration of LA extract generates other impurity phases also. It is also observed that the degree of crystallinity is first increasing slightly and then decreasing as the concentration of LA content is increased. This can be due to the formation of Zn(OH)_2_ impurity phase. The obtained results are in well agreement with earlier report.^56^ Hence, the degree of crystallinity and size of the crystallites are significantly influenced with the amount of LA extract used in the synthesis process.

#### 3.3.3 Effect of calcination temperatures

**Fig. 14:**
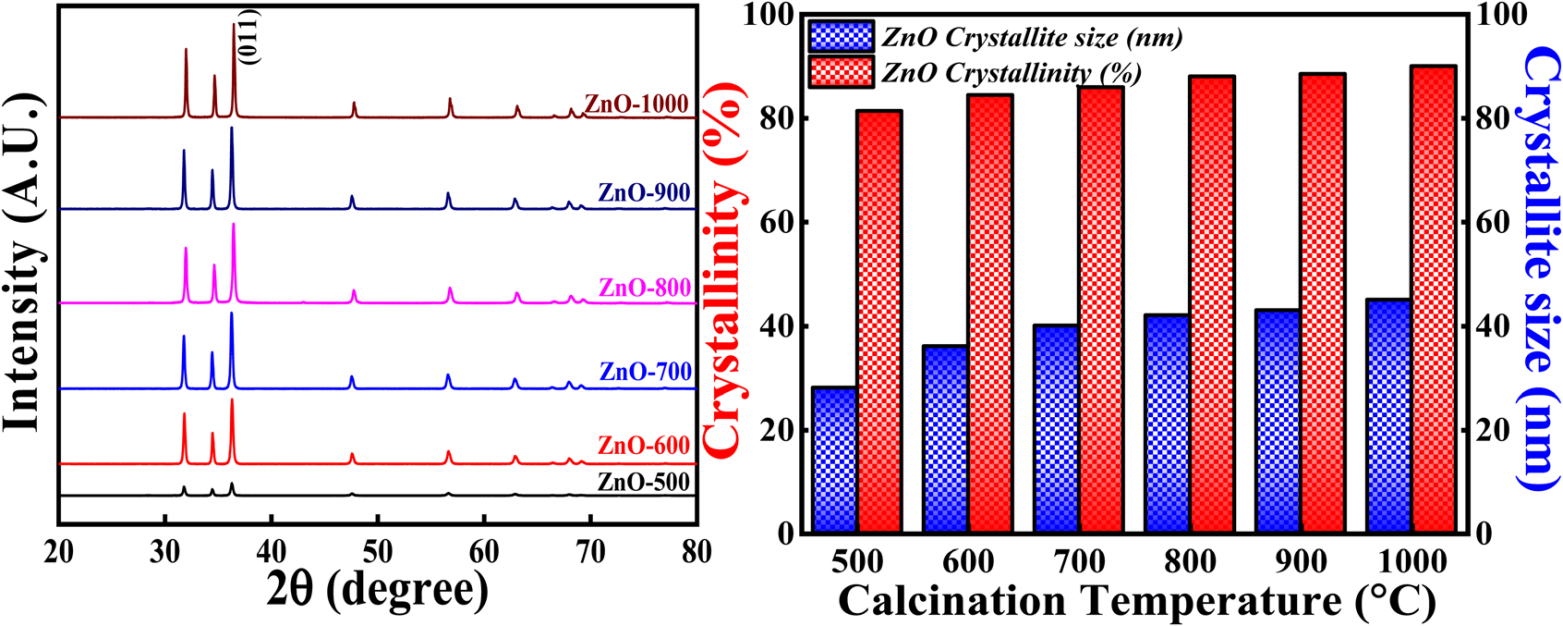
(Left) XRD patterns, (Right) crystallinity and crystallite size studies based on calcinations temperature.

Fig. 16 (Left) represents the XRD profiles of ZnONPs calcined at different temperatures *i.e.*,500 °C, 600 °C, 700 °C, 800 °C, 900 °C and 1000 °C. Fig. 14 (Right) shows the crystallite size and crystallinity percentage pattern with respect to different calcinations temperatures. All the samples calcined at different temperatures show single phase hexagonal crystalline structure without forming any intermediate phases (ICSD ref. code: 98-008-1255). From the XRD pattern, it is clearly observed that the intensity of each peak increases with the increase in the calcinations temperatures. The slight increment in the peak intensity of (011) plane is depicted in closure picture of Fig. S4.† These results clearly indicate that the degree of crystallinity is increasing with the increase in the calcinations temperature. The highest crystallinity percentage is 90.04% which is obtained for ZnO-1000. Moreover, there is a slight variation in the Bragg angle for (011) plane (Fig. S3†) as the calcinations temperatures is varied, suggesting that the calcinations temperature would affect the atoms' orientation in lattice planes. The higher calcinations temperature will provide more energy for the atoms to diffuse and occupy at particular site in the crystal lattice.^57^ The change in Bragg angle of (011) plane can be related to stress factor as discussed in.^58^

Similarly, the crystallite size showed increasing trend as the calcinations temperature is increased. But the crystallite size increased more from 500 °C to 700 °C and then slight increment from 700 °C to 1000 °C. The more increment in the crystallite size from 500 °C to 700 °C can be due to the coalescence process.^47,48^ This process can cause major crystal and grain growth enhancing surface roughness and porosity. The later slight increment in the crystallite size from 700 °C to 1000 °C can be due to the higher mobility of atoms leading them to agglomerate. Sometimes, random motion of the atoms follows dispersion and diffusion as well. As a result, all the factors compete with each other in order to determine the crystallite size. Generally, the increase in the crystallite size can be related to the merging of particles as the consequence of thermal calcination. Hence, the calcinations temperature plays a vital role in tuning the crystallite size and degree of crystallinity.

#### 3.3.4 Effect of calcinations hours

**Fig. 15:**
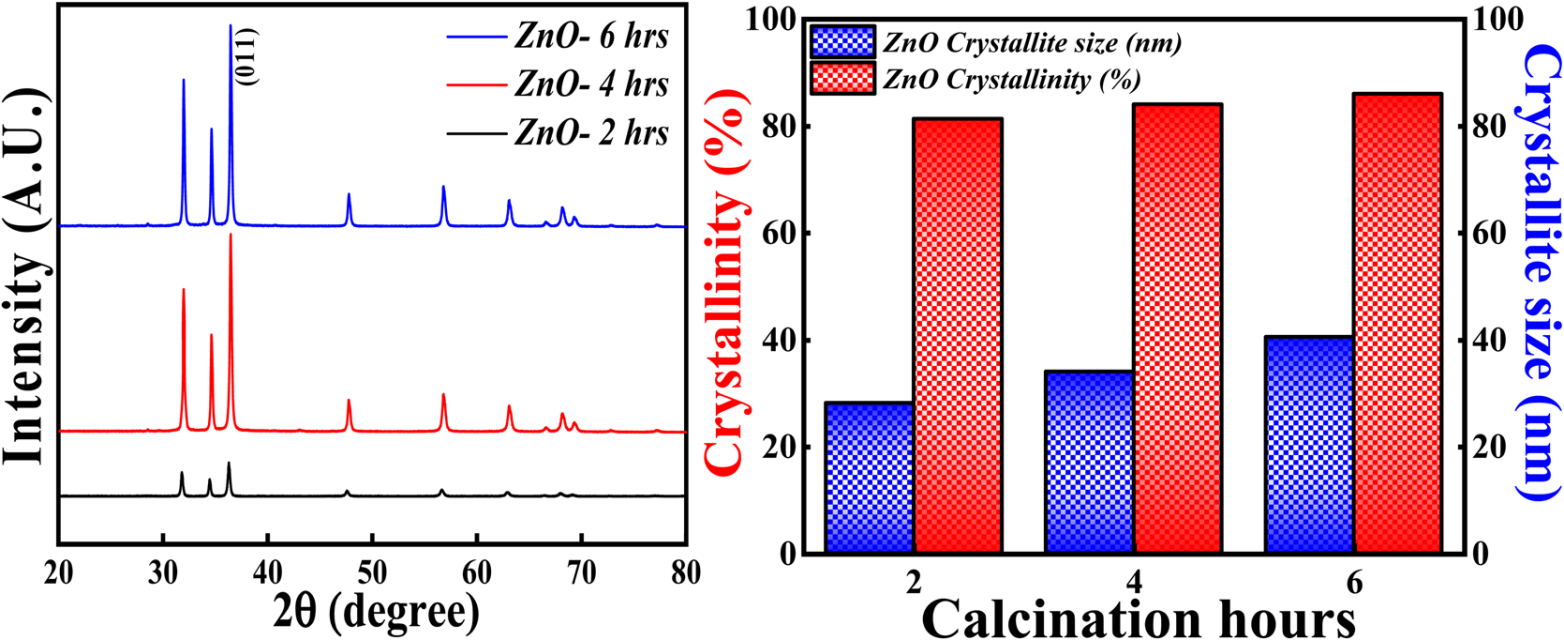
(Left) XRD patterns, (Right) crystallinity and crystallite size studies based on calcinations hours.

The XRD analysis was carried out for ZnO NPs synthesised at different calcinations hours (2, 4 and 6 hours) and the obtained XRD pattern along with crystallinity and crystallite size results are depicted in Fig. 15. All the samples; ZnO-2 h, ZnO-4 h and ZnO-6 h showed single phase hexagonal wurtzite crystalline structure (ICSD ref. Code: 98-008-1255). There are no additional peaks for all the samples, which means that there are no foreign particles or any secondary (mixed) phases as the calcinations hour is changed. The calculated crystallinity percentage and crystallite size are 81.40%,83.90% and 86.01%, 28.12 nm, 34.01 nm and 40.50 nm respectively. These results clearly specify that the crystallinity is slightly increasing with the increase in the calcinations hours. The enhancement in the degree of crystallinity is shown in Fig. S4† as there is a sequential increment in the peak intensity of (011) plane. Similarly, the crystallite size is systematically increasing with the increase in the calcinations hours. This can be due to the agglomeration of crystallites as more calcinations time facilitates the crystal growth. These results are in good agreement with the previously reported literature pertaining to effect of calcinations time.^59^ So, the calcinations hours are slightly affecting the degree of the crystallinity but having a significant effect on crystallite size.

The main observation from the XRD results is that the degree of crystallinity and crystallite size can be tuned based on the concentration of precursors, leaf extract, calcinations temperatures and calcinations hours. The appropriate sample can be used for the effective decomposition of MB dye based on their properties.

### 3.4 Photocatalytic studies

#### 3.4.1 Mechanism of photodegradation of MB dye

**Fig. 16:**
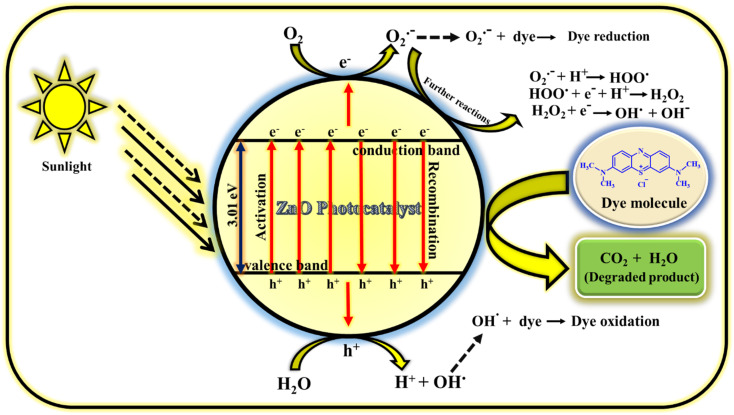
Mechanism of photocatalytic performance of ZnO NPs using MB dye.^60^

The photodegradation of MB dye using ZnO photocatalyst follows radical generation mechanism. The radicals generated in the solution are highly reactive and attacks the dye which results in less harmful products (carbon dioxide and water). The possible way of radical generation with the help of ZnO photocatalyst is clearly illustrated in the Fig. 16. When the sunlight is irradiated on the MB dye solution containing milligram of ZnO catalyst, electrons (e^−^) and holes (h^+^) are produced in the conduction and valence bands of photocatalyst.^61^ This e^−^ and h^+^ act as charge carriers for the generation of radicals. The e^−^ in the valence band reacts with atmospheric or adsorbed oxygen to give O_2_˙^−^ (oxygen radical anion or superoxide anion) and h^+^ in the valence band reacts with water molecules to give H^+^ (proton) and OH˙ (hydroxyl radical). Similarly, the h^+^ also reacts with adsorbed hydroxyl anion (OH^−^) to give OH^.^^62^ These radicals may react further to give corresponding reactive oxygen species radicals such as HOO˙ (peroxide radical), OH˙ and OH^−^ (hydroxyl anion). Generally, the oxygen radical anion and hydroxyl radical attack and degrade the dye molecules by undergoing reduction and oxidation process with the dye molecules. But other radicals generated as the consequence of further reactions of O_2_˙^−^ also helps in the degradation process. So, the prime reason for the dye decomposition is the radical's generation (O_2_˙^−^, OH˙, HOO˙,˙OH^−^). Even though it is the prime factor for dye degradation but there are some other secondary factors also in which the dye decomposition depends. They are; reaction time, catalyst loading, precursor concentration, pH conditions, oxidants, calcinations temperature, calcinations hours, doping *etc.* In the present study, reaction time, catalyst loading, precursor concentration, calcinations temperature and calcinations hours are the parameters taken into consideration which have given the excellent result without including the external factors such as pH conditions, oxidants, doping. The prime reason for not including these external factors is the concept of focusing on green chemistry. The detailed study on photocatalytic activity of ZnO NPs for the dye degradation is explored by focusing on various parameters. Hence, if all the parameters deciding the dye degradation is optimised, water pollution can be minimised using this catalyst.

#### 3.4.2 Effect of ZnO photocatalyst loading

**Fig. 17:**
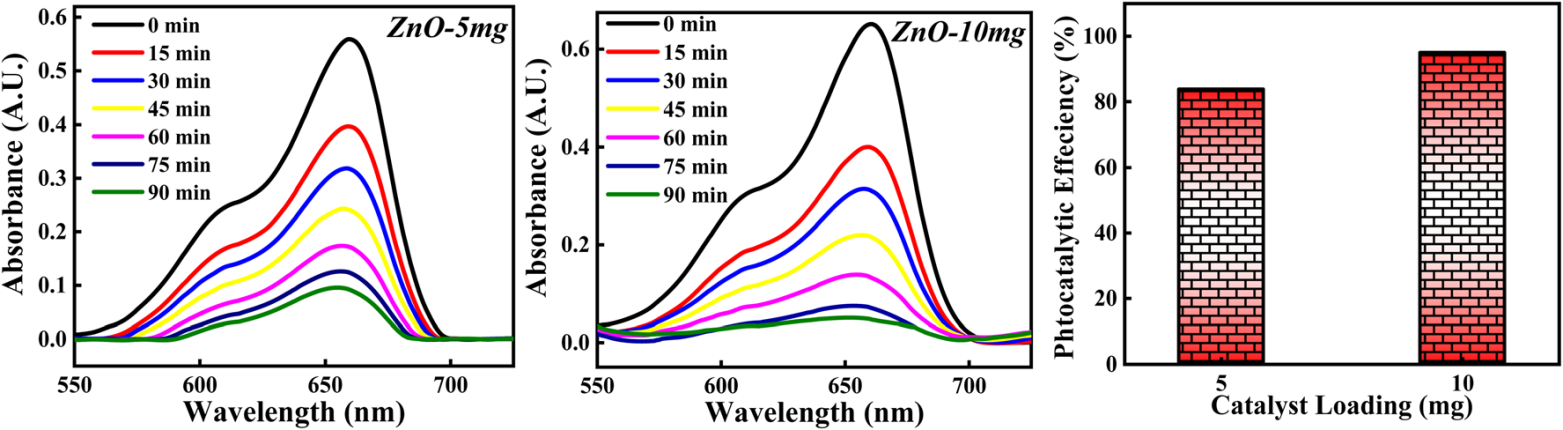
The photodegradation of MB dye based on 5 mg and 10 mg ZnO photocatalyst.

The photocatalytic potency of ZnO catalyst based on catalyst loading for 10^−5^ M MB dye solution was studied for 5 mg and 10 mg photocatalyst calcined at 500 °C and the photocatalytic plots are shown in Fig. 17. The photocatalytic efficiency calculated for 5 mg and 10 mg is 84.10% and 95.01%. It is clearly observed that the percentage of degradation of MB dye is increased as the amount of catalyst is increased. This can be due to more adsorption of photons by the catalyst surface resulting in a greater number of e–h pairs which can generate more radicals. Larger amount of catalyst can generate more active sites available for adsorption of dye molecules as well as adsorption of OH^−^. All these factors will favour the radical generation which can degrade the dye efficiently. But as the amount of catalyst is increased beyond a limit, there are some other factors which can decrease the photocatalytic efficiency of ZnO NPs. The possible reason is that high loading of catalyst results in increased turbidity of solution which leads to lesser penetration of solar rays into the solution. Moreover, increase in the amount of catalyst causes overcrowding of adsorbent species results in overlapping of active sites for adsorption.^63^ Also, it can cause collision between the active adsorbed species with the ZnO without any adsorption taken place resulting in the deactivation of ZnO catalyst. Hence, catalyst loading to degrade the dye should be optimised in such a way that the highest photocatalytic efficiency is achieved.

#### 3.4.3 Effect of concentrations of MB dye

The photodegradation of MB dye by varying the MB dye concentration from 10^−5^ M to 10^−4^ M is studied without changing the ZnO loading. The photocatalytic results obtained are shown in Fig. S11.† It is noticed that as the concentration of dye is increased, the photocatalytic activity of 5 mg ZnO photocatalyst is decreased. This can be due to the following reasons; firstly, as the concentration of dye is increased, the adsorption of dye molecules on the active sites of photocatalyst would ultimately increase. So, the deactivation of ZnO semiconductor will be more as the irradiation of photocatalyst with solar light will be less. As a result, the generation of e–h pairs will be very less. Moreover, more adsorption of dyes molecules decreases the adsorption of OH^−^which suggests that the rate formation of formation of OH˙ will be very less.^64^ Secondly, increase in the concentration of dye decreases the penetration of solar rays into the reaction mixture.^20^ Ultimately, the generation of radicals decreases. Hence, the particular amount of ZnO will degrade only the specific or lesser concentration of dye.

#### 3.4.4 Effect of concentrations of Zn(NO_3_)_2_·6H_2_O

**Fig. 18:**
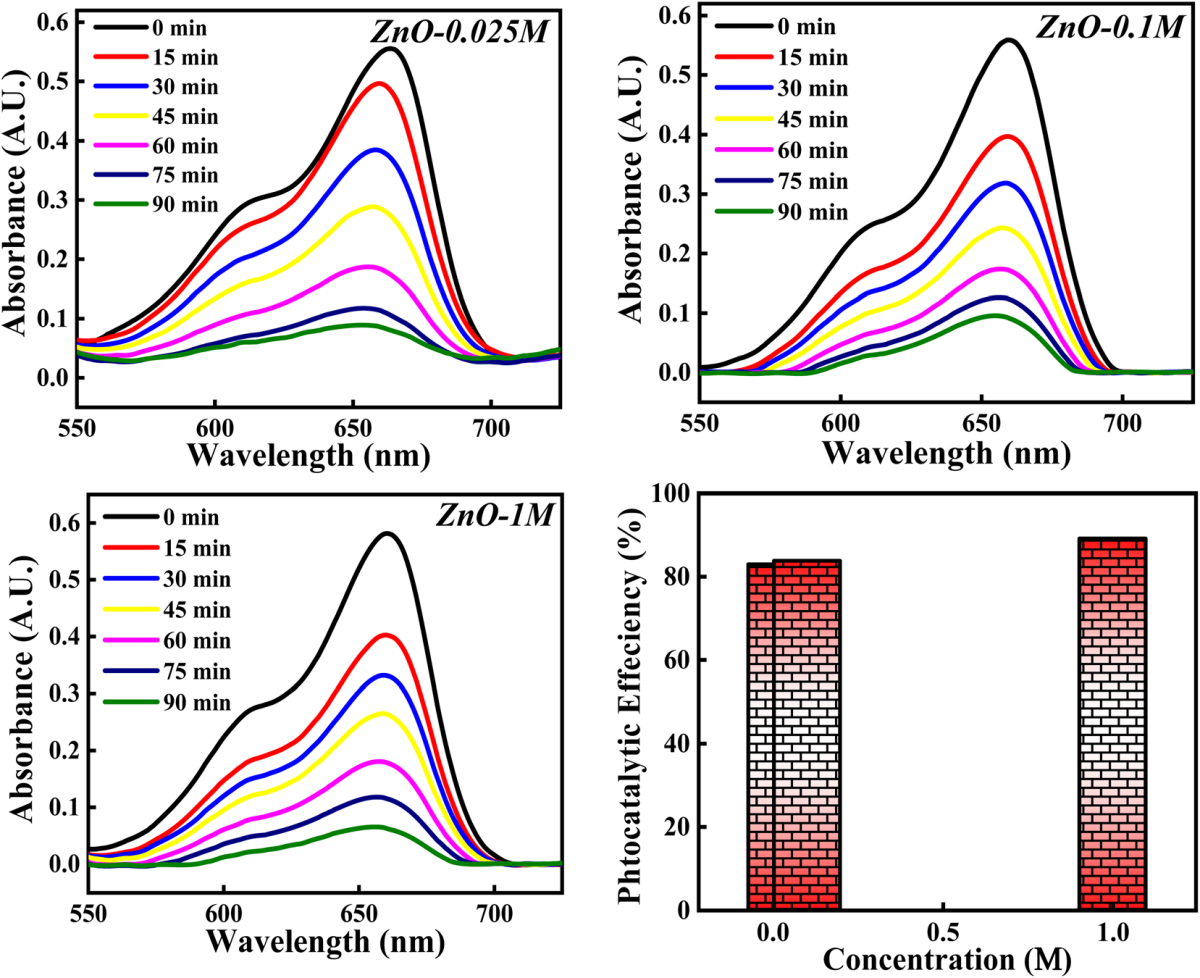
The photocatalytic activity of ZnO NPs based on concentration of Zn(NO_3_)_2_·6H_2_O.

The photocatalytic potency of ZnO NPs for the decomposition of MB dye is evaluated based on three precursor concentrations of Zn(NO_3_)_2_·6H_2_O *i.e.* 0.025 M, 0.1 M and 1 M and the obtained photocatalytic plots are shown in Fig. 18. The photocatalytic efficiency calculated for 0.025 M, 0.1 M and 1 M concentrations of precursor are 82.99%, 84.10% and 89.12%. It is interesting to note that the photocatalytic degradation of MB dye increases with the increase in the precursor concentration from 0.1 M to 1 M. However, for 0.025 M and 0.1 M concentration, the photocatalytic efficiency is slight varied. This can be due to the same band gap values obtained for 0.025 M and 0.1 M concentration (Fig. S5†) which results in similar result. But 1.11% increment in efficiency can be due to other factors such as good crystallinity which has resulted in generation of more e–h pairs for radical generation.

There is a slight variation of in band gap values (3.00–3.01 eV) for 0.025 M, 0.05 M and 0.1 M concentration was observed, as depicted in Fig. S6.† Above the 0.1 M concentrations, the band gap was not able to calculate which can be due to the poor dispersion of the particles in solution as a result of agglomeration for the higher concentration. Similarly, total surface area calculated from BET didn't show much variation, which can reflect in the photocatalytic results (Fig. S7†). The 5.02% increment in the photocatalytic efficiency for 1 M from 0.1 M concentrations can be due to the defect generation (O_v_, Zn_i_) which can introduce defect level in between the conduction and valence bands. This impurity level can act as trappers for charge carriers which can enhance the recombination process^65^ and thus improving the degradation process. Moreover, as the particle size increases, band gap decreases (quantum size effect). Hence, the photocatalytic activity of ZnO NPs catalyst for the degradation of MB dye depends on the precursor concentrations as well.

#### 3.4.5 Effect of concentration of LA extract

**Fig. 19:**
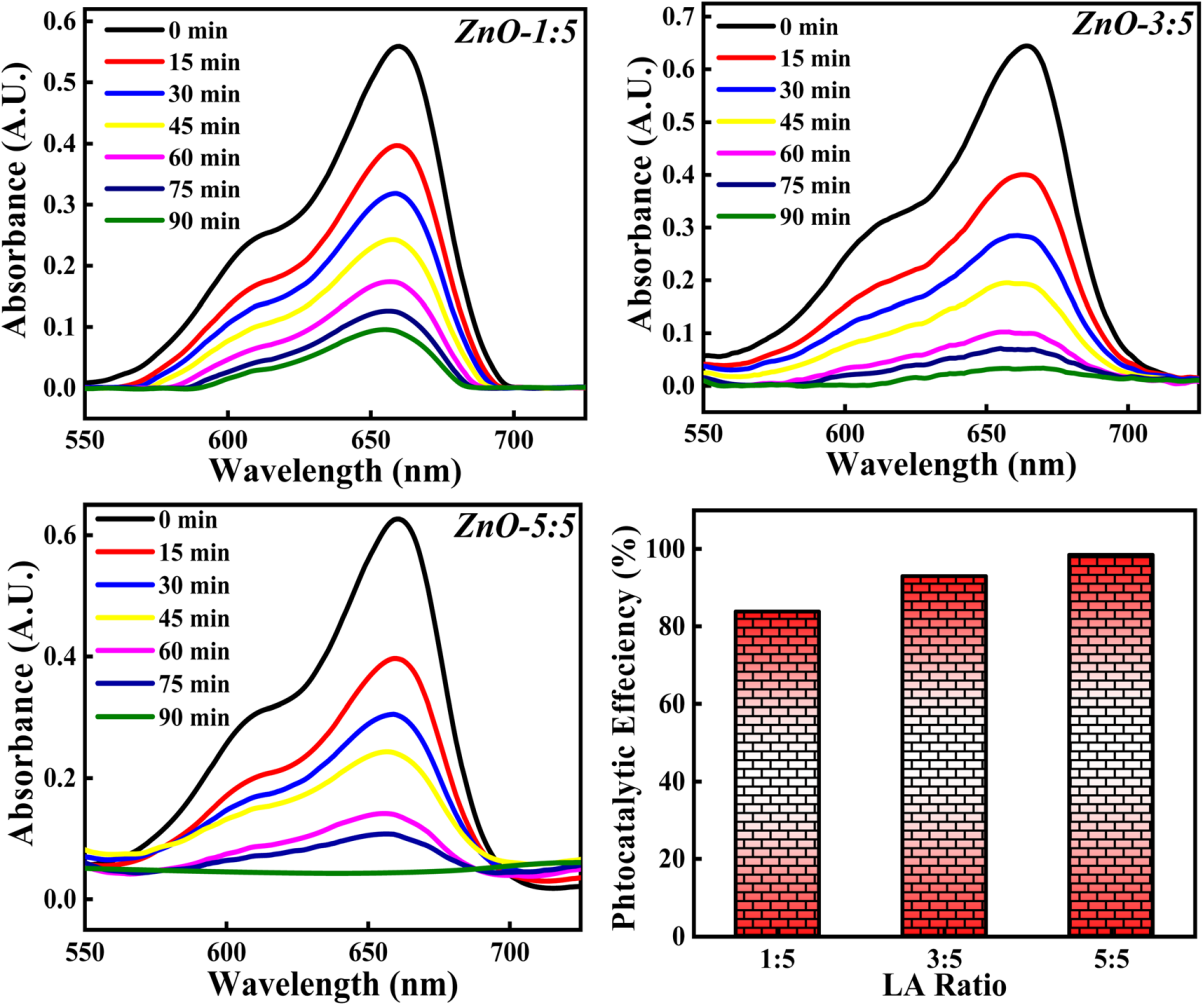
The effect of concentrations of LA extract on the photocatalytic degradation of MB dye.

The photocatalytic degradation of MB dye based on the concentration of LA extract (5 mL, 10 mL and 25 mL) is explored and the obtained photocatalytic results are represented in Fig. 19. The photocatalytic efficiency calculated for ZnO-1 : 5, ZnO-3 : 5 and ZnO-5 : 5 is 84.10%, 92.36% and 98.50%. It is clearly noticed that the photocatalytic potency is increasing significantly with the increase in the concentration of LA extract. This significant increase in the photocatalytic degradation of MB can be due to various reasons such as Zn(OH)_2_ impurity phase generation, small size crystallites, higher surface area, enhanced recombination process *etc.* From the XRD results, it is clearly observed that the crystallite size decreases and the concentration of Zn(OH)_2_ increases as the LA extract concentrations are increased. Firstly, the presence of Zn(OH)_2_ can introduce an impurity level below the conduction band, which can act as scavengers of charge carriers enhancing the recombination time,^66^ thus reducing the band gap. Moreover, the presence of surface hydroxyl group, facilitate the formation of non-covalent bond with the dye molecules. Thus, enhancing the adsorption can improve the photocatalytic process. Secondly, as the concentration of LA is increased, the crystallite size is decreased confirming the presence of small size particles providing higher surface area for adsorption of dye molecules. Thirdly, the presence of hydroxyl group on the catalyst surface can produce OH˙, enhancing the photocatalytic decomposition of MB dye.^12^ So, the factors such as presence of OH group, small size crystallites, and high surface area enhance the photocatalytic process. So, the concentration of LA extract should be optimised in order to get small size ZnO particles for 100% MB degradation, without generating any impurity phases such as Zn(OH)_2_. Hence, the concentration of LA extract is greatly influencing the dye degradation process.

#### 3.4.6 Effect of calcinations temperatures

**Fig. 20:**
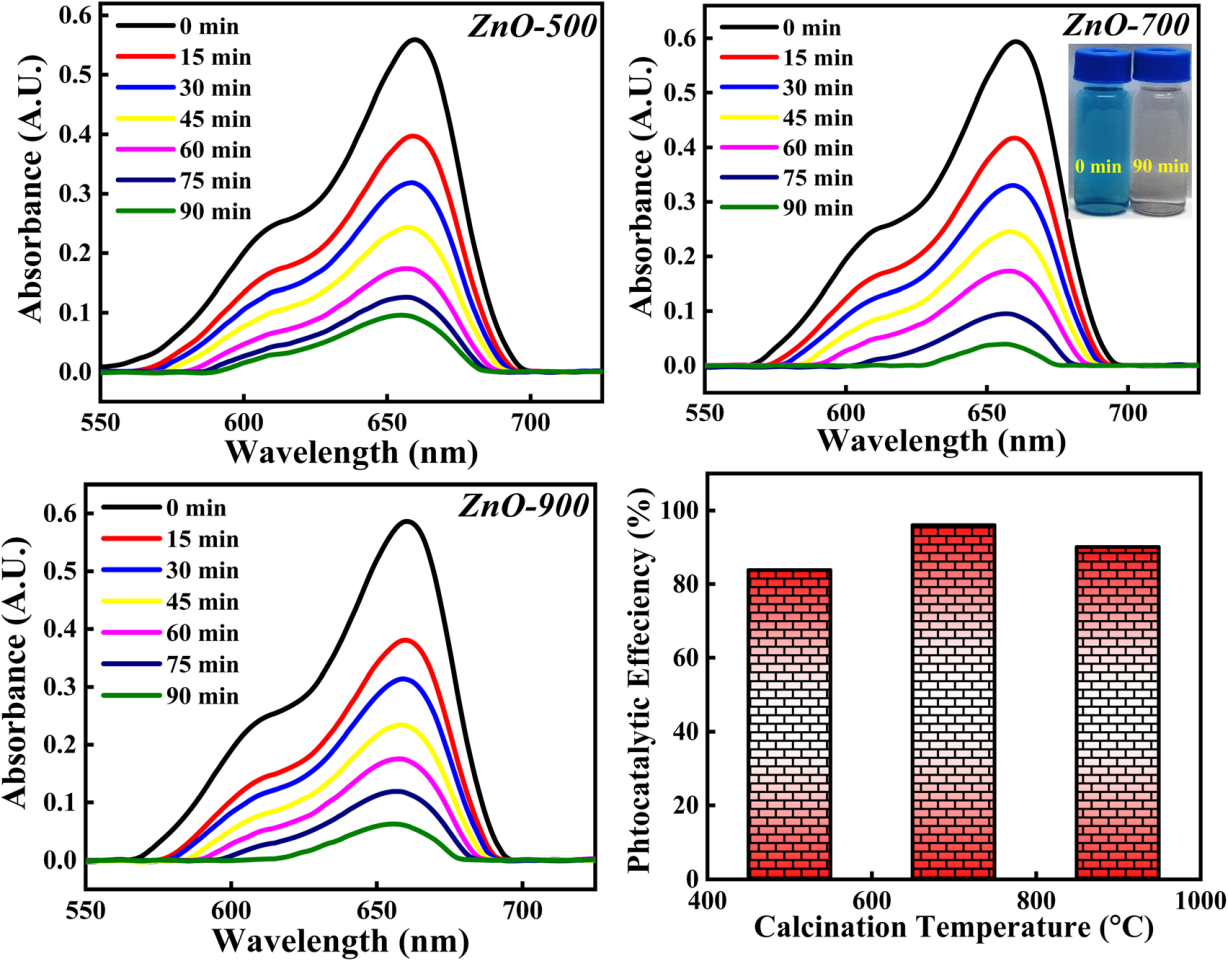
The photocatalytic degradation of MB dye based on three calcinations temperatures (500 °C, 700 °C and 900 °C).

The photocatalytic degradation of MB dye is analysed with respect to ZnO-500, ZnO-700 and ZnO-900 samples and the obtained photocatalytic results are depicted in Fig. 20. The photocatalytic activity percentage calculated for ZnO-500, ZnO-700 and ZnO-900 is 84.10%, 96.02% and 90.06%. It is observed that the photocatalytic efficiency increases as the calcinations temperatures is increased from 500 °C to 700 °C and then decreases when the calcinations temperature is further increased to 900 °C. The initial increment in the photocatalytic efficiency can be due to reduction in the band gap (as represented in Fig. S6†). The increase in calcinations temperature may generate more defects as well as results in agglomeration of particles. These defects reduce the band gap by introducing impurity level above the valence band as discussed earlier. Moreover, the agglomeration of particles reduces the band gap because of the principle of quantum size effect. Hence, both factors; generation of defect and particle agglomeration are narrowing the band gap which improves the recombination process and thereby improving the photocatalytic degradation activity. The later decrement in the photocatalytic activity can be due to the particle agglomeration, even though the band gap is reducing. The dominance of one factor over the other will reflect in the photocatalytic results. The particle agglomeration may be dominant over the band gap reducing factor which would have lowered the photocatalytic efficiency percentage. Moreover, increase in the calcinations temperature may favour sintering process also, which will deactivate the photocatalyst by decreasing the surface area, pores and thus eliminating the catalytic sites of the ZnO photocatalyst.^67^ So, the photocatalytic degradation of MB dye greatly depends on the calcinations temperature but doesn't follow general trends. There are several other factors which decide the degradation percentage along with the calcinations temperature. Hence, the optimised calcinations temperature should be considered for the complete degradation of MB dye.

#### 3.4.7 Effect of calcinations hours

**Fig. 21:**
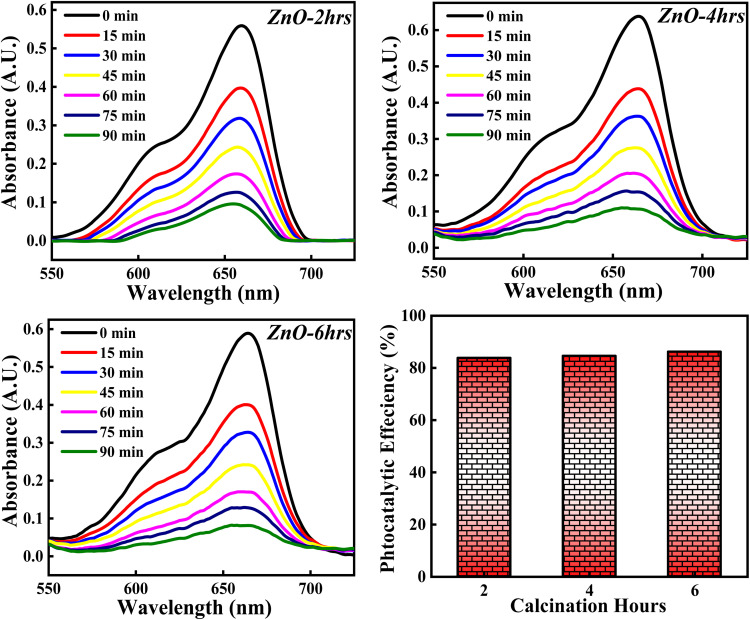
The influence of calcinations hours (2, 4, 6) on % degradation of MB dye.

The photocatalytic activity of ZnO NPs catalyst on percentage degradation of MB dye is examined with respect to 2, 4 and 6 hours of calcinations and the obtained photocatalytic plots of ZnO-2, ZnO-4, and ZnO-6 are depicted in Fig. 21. The photocatalytic potency percentage calculated ZnO-2, ZnO-4, and ZnO-6 are 84.10%, 84.70% and 86.05% respectively. These results signify that increase in the calcinations hours doesn't produce any significant change on the decomposition of MB dye. The small increment in the catalytic efficiency may be due to good crystallinity resulting in more generation of e–h pairs as a result of more adsorption of photons. Even though a small increment in photocatalytic activity is observed, it was not significant. Hence, the tuning of calcinations hours does not play any significant role towards the photocatalytic degradation of MB dye.

A comparative study of photocatalytic efficiency of ZnO NPs of current work with the previous results is represented in Table 3.

**Table 3:** Comparative study of photocatalytic efficiency of ZnO NPs of present work with the previous results^68–71^

Sample name	Synthesis method	Light source	% Efficiency (time)	Photocatalyst/MB concentration	Reference
M_*x*_Zn_1−*x*_O (M = Al^3+^, Fe^3+^, Cr^3+^)	Chemical method	Visible light	91% (120 min)	—	68
PQ/ZnO	Chemical method	Sunlight	80% (90 min)	50 mg/10 ppm	69
ZnO	Chemical method (autoclave)	Mercury vapor lamp	92% (60 min)	50 mg/10 ppm	70
ZnO NPs	Green method-*Sambucus ebulus* extract	UV light	80% (200 min)	20 mg/50 ppm	71
ZnO NPs	Green method-LA leaf extract	Sunlight	98.5% (120 min)	5 mg/10 ppm	Present work

## 4. Conclusion

The green approach towards the synthesis of ZnO NPs using a novel LA leaf extract offered a simple, stable, economically viable, less invested, sustainable and eco-friendly method. The systematic study of ZnO NPs showed diverse morphologies, low band gap (2.81–3.01 eV) with increase in calcinations temperatures, and generation of Zn(OH)_2_ with the increase in LA concentration. The crystallinity percentage and crystallite size were significantly affected with the variation in concentration of precursor, concentration of LA leaf extract, calcinations temperatures and calcinations hours which played a crucial role in the activity of ZnO NPs as a photocatalyst. The mentioned synthesis process can be employed in industrial scale so that waste minimisation can be increased as well as enhancing simple high yield production. To be employed in industrial scale, all the synthesis parameters should be optimised in such a way that ZnO NPs can be accumulated in a larger amount. The photocatalytic application of ZnO NPs showed an excellent result by degrading the MB dye completely. The dye degradation study was done systematically based on various physicochemical parameters which guided towards the efficient photocatalytic result, 96–98.5% (5 mg degrading 10 ppm MB dye completely). Hence, ZnO NPs photocatalyst synthesised through systematic green chemistry protocol can be used effectively for degrading other toxic dyes and can be employed in large-scale for the effective treatment of waste water.

## Author contributions

Supin K. K: writing – original draft, methodology. Formal analysis, investigation, conceptualization, validation and visualization, Parvathy Namboothiri P M: partial analysis and visualization, M. Vasundhara: conceptualization, writing – review & editing, visualization, supervision, project administration and funding acquisition.

## Conflicts of interest

The authors declare that there is no conflict of interest regarding the publication of this article.

## Supplementary Material

RA-013-D2RA06967A-s001
