# Supplementary material for: Enhanced photocatalytic activity in ZnO nanoparticles developed using novel Lepidagathis ananthapuramensis leaf extract
Source: RSC Adv. 2023 Jan 6;13(3):1497–515. doi: 10.1039/d2ra06967a (PMC9819108; doi:10.1039/d2ra06967a)
Supplement: RA-013-D2RA06967A-s001 [file RA-013-D2RA06967A-s001.pdf]

Electronic Supplementary Material (ESI) for RSC Advances.
This journal is © The Royal Society of Chemistry 2022

Supplementary information

Enhanced Photocatalytic Activity in ZnO Nanoparticles Developed Using Novel *Lepidagathis Ananthapuramensis* Leaf Extract

Supin K K[a], Parvathy Namboothiri P M[a] and M Vasundhara[a,b*]

[a]*Polymers and Functional Materials Department, CSIR- Indian Institute of Chemical Technology, Hyderabad-500007, India.*
[b]*Academy of Scientific and Innovative Research (AcSIR), Ghaziabad-201002, India.*

**Corresponding author: mvas@iict.res.in*

1. Phytochemicals detection

Method

About 50g of fresh leaves of LAwere weighed and ground into powdered form. The powder was soaked overnight in 300ml methanol. The methanolic extract was then filtered using standard filter paper and allowed to evaporate the methanol from the filtered extract. The remaining methanol from the extract is evaporated and the plant extract is concentrated with the help of a rotary evaporator, the concentrated plant extract left in the round bottomed flask was collected using a minimum quantity of acetone or methanol. Then methanolic plant extract was tested for the presence of different phytochemicals as follows.

Preliminary phytochemical analysis

Test for alkaloids

3.0 ml of crude extract was stirred with 3.0 ml of HCl on the steam bath, few drops of Dragendroff's reagent were further added in the test tube and the occurrence of orange red precipitate was found, which is taken as positive test.

Test for flavonoids

Shinoda test: The crude extract was mixed with few fragments of magnesium ribbon and then concentrated HCl was added drop wise. The pink scarlet colour that appeared after few minutes indicates the presence of flavonoid.

Test for saponins

The crude extract was added to 5ml of distilled water in a test tube. The solution was shaken vigorously and observed for a stable persistent froth, which indicates the presence of saponins.

Test for terpenoids and steroids

Salkowski's test: 1.0 ml of the extract was taken in a boiling tube and 2.0 ml of concentrated sulphuric acid was added slowly and red violet colour was observed for terpenoid and green bluish colour for steroid.

Test for tannins

To 1.0 ml of extract solution, 1-2 drops of lead acetate solution was added. Red precipitate formation indicates the presence of tannins.

Test for phenols

Ferric chloride test: 1.0 ml of the extract was taken in a test tube and added with few drops of neutral 5% ferric chloride solution. Dark green colour indicates the presence of phenolic compound.

Test for glycosides

To the solution of the extract, few drops of sodium hydroxide were added and observed for yellow colour, which shows the presence of glycosides.

Test for cardiac glycosides

5.0 ml of the extract was taken in a boiling tube to which 2.0 ml of acetic acid containing one drop of ferric chloride solution was added and 1ml of conc. Sulphuric acid was added slowly. The appearance of brown ring indicates the presence of cardiac glycosides.

Test for reducing sugars

The crude extract was shaken with 5.0 ml of distilled water and filtered. The filtrate was boiled with drops of Fehling's solution A and B for 2 minutes. An orange red precipitate indicates the presence of reducing sugars.

Test for proteins

By adding 1ml of 40% NaOH and few drops of 1% copper sulphatewas added to 2.0 ml of the extract. Nocolourchange was found indicatedthe peptide linkage of molecule is absent in the extract.

Test for quinones

1.0 ml of the extract was added to the concentrated HCl. The formation of yellow precipitate or coloration indicates the presence of quinones

Phytochemical screening of leaf extract of *Lepadagathis Ananthapuramensis*

**Phytochemicals**	**Presence (+)/ Absence (-)**
Alkaloids	+
Flavanoids	+
Saponins	+
Tannins	+
Terpenoids	+
Glycosides	+
Cardiac glycosides	+
Proteins	-
Reducing sugars	+
Quinones	+
Phenols	+

2. SEM analysis:

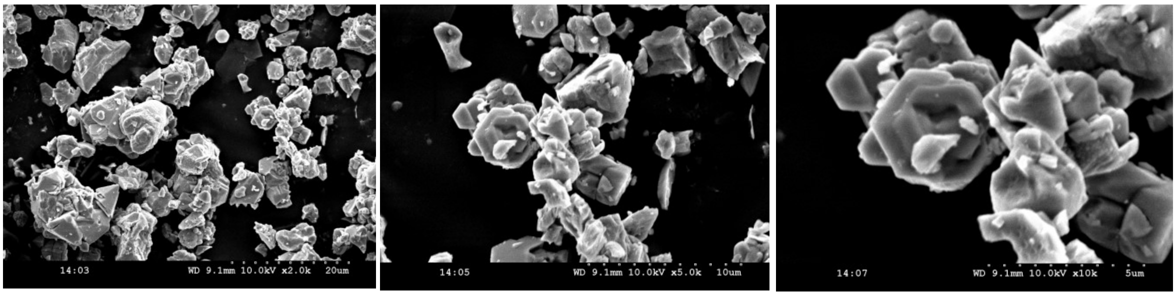

Figure S1: SEM micro images of ZnO NPs

3. HR-TEM analysis:

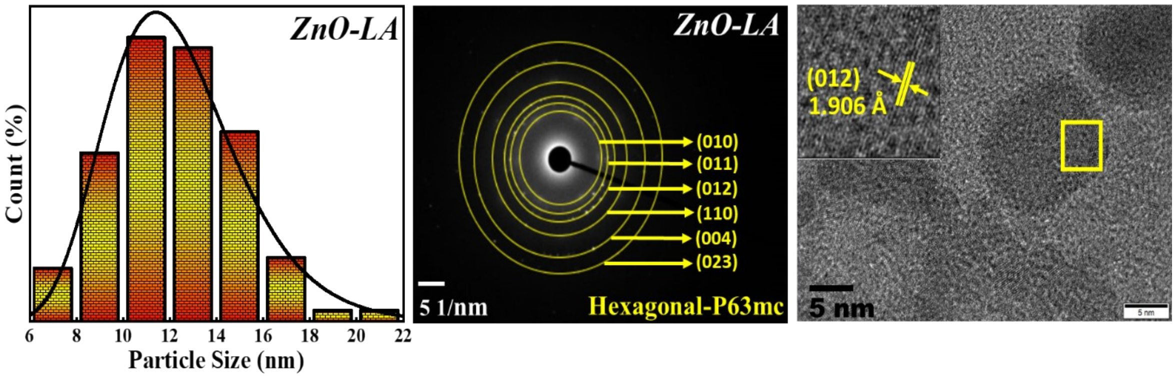

Figure S2: Histogram, SAED and lattice fringes patterns of ZnO NPs

3. DLS analysis:

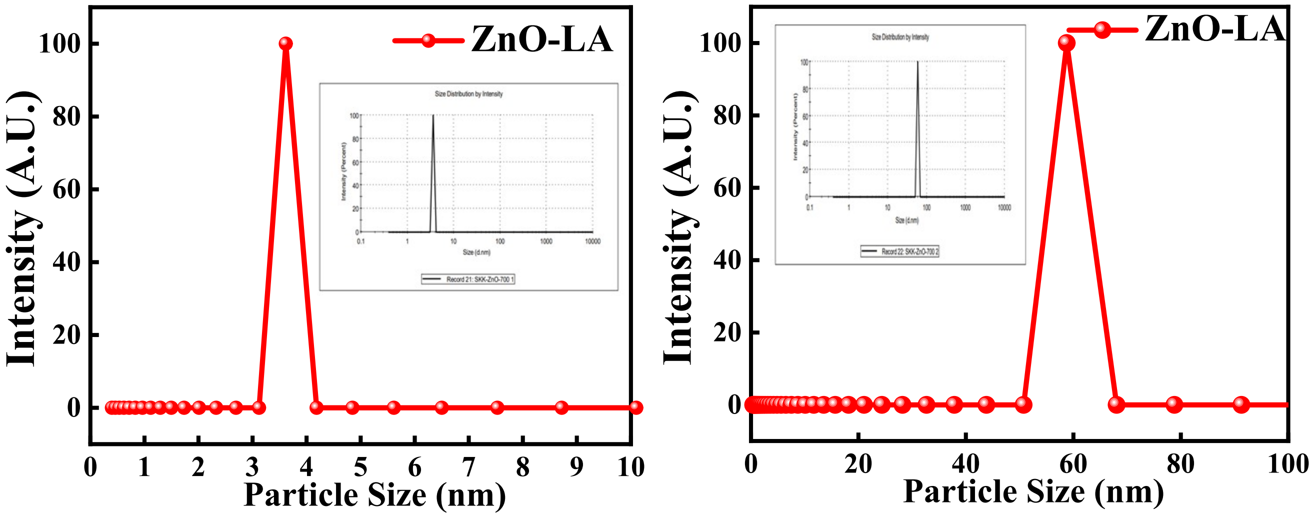

Figure S3: DLS plots of ZnO NPs

4. XRD analysis:

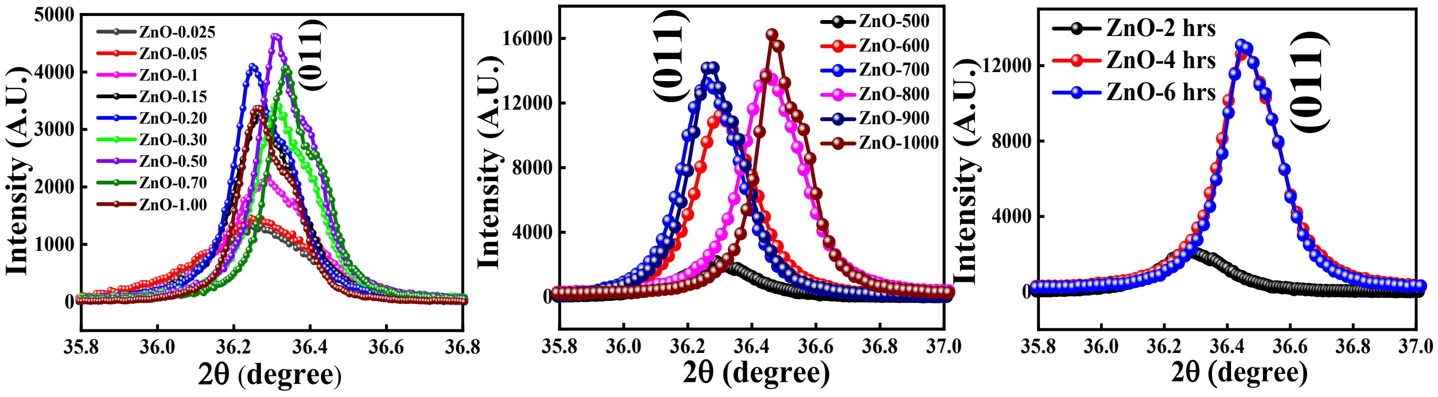

Figure S4: (Left) XRD patterns: Closure plots of (011) plane based on concentrations of precursors, (Middle) calcination temperatures and (Right) calcination hours

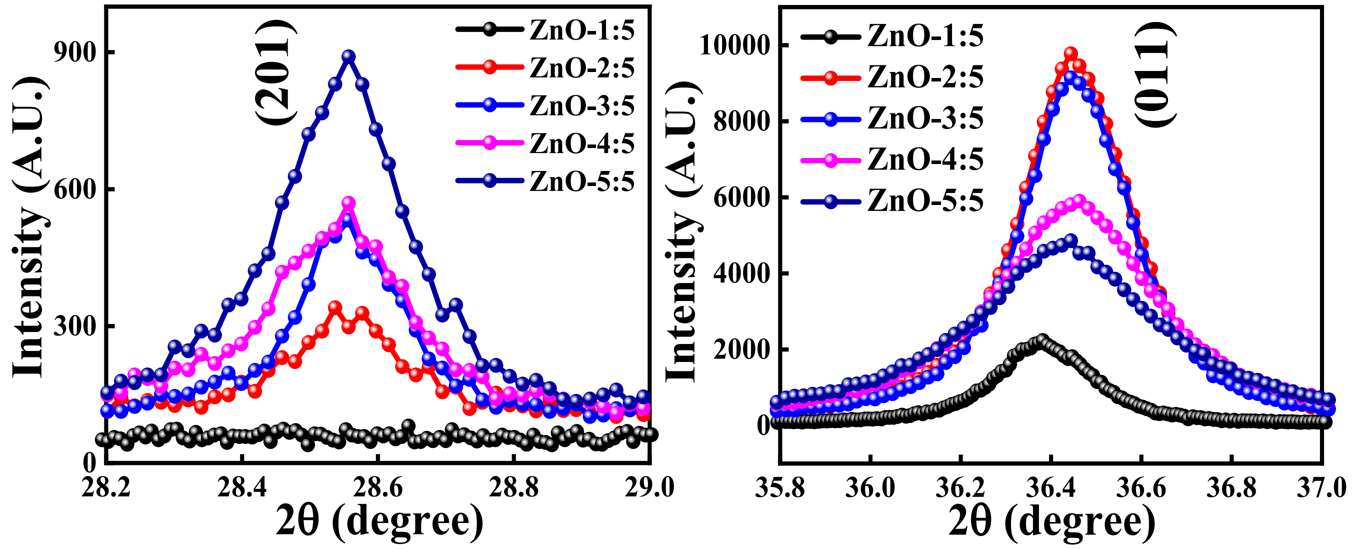

Figure S5: XRD patterns: Closure plots of (201) and (011) planes based on concentration of LA extract

5. UV-Tauc plot analysis

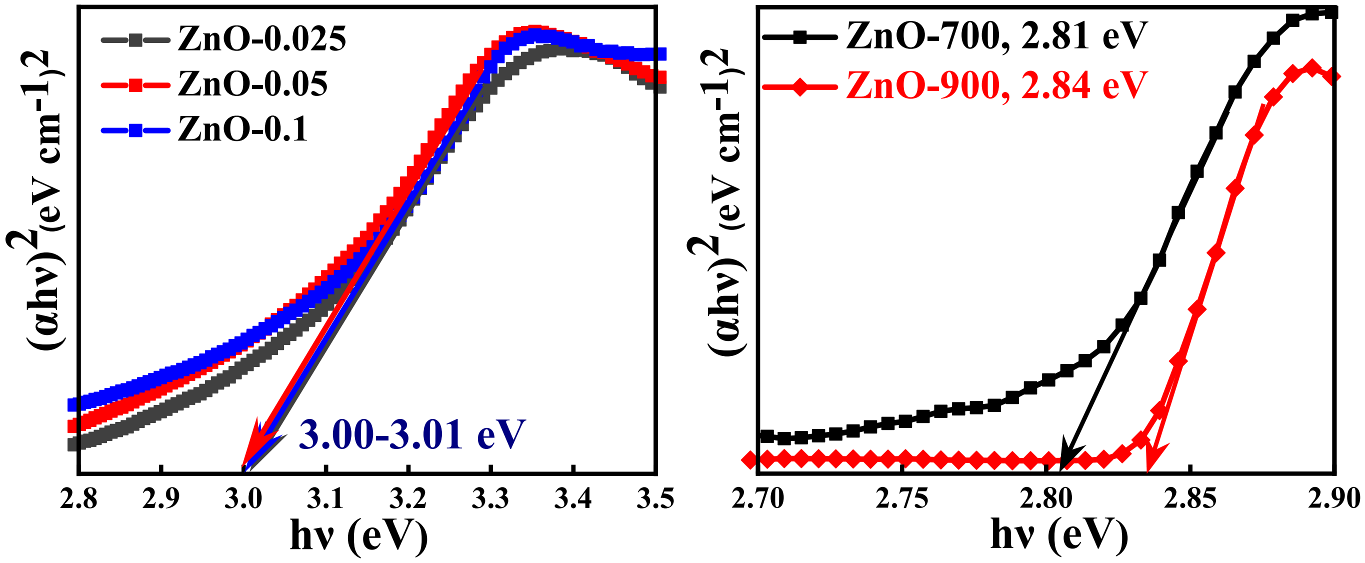

Figure S6: Tauc plot based on effect of concentration of $Zn(NO_3)_2.6H_2O$ and calcinations temperatures

6. BET plots

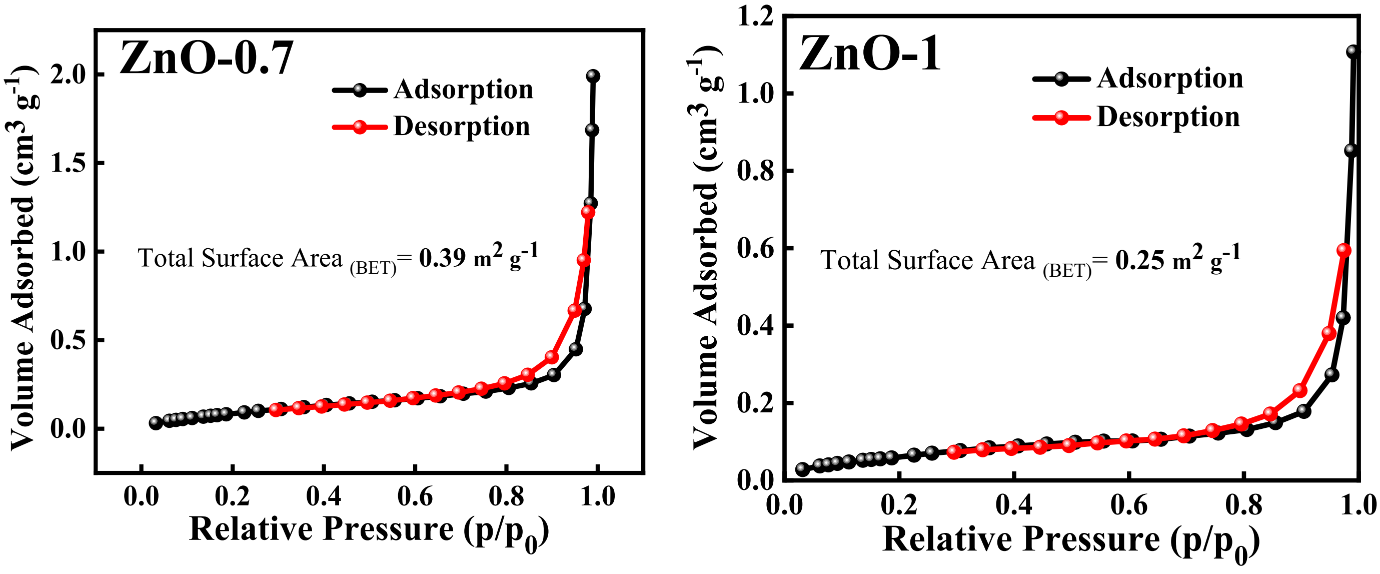

Figure S7: N_2 Adsorption and desorption isotherms showing BET surface area of ZnO NPs

7. Photocatalytic studies

7.1 Importance of photocatalyst and sunlight

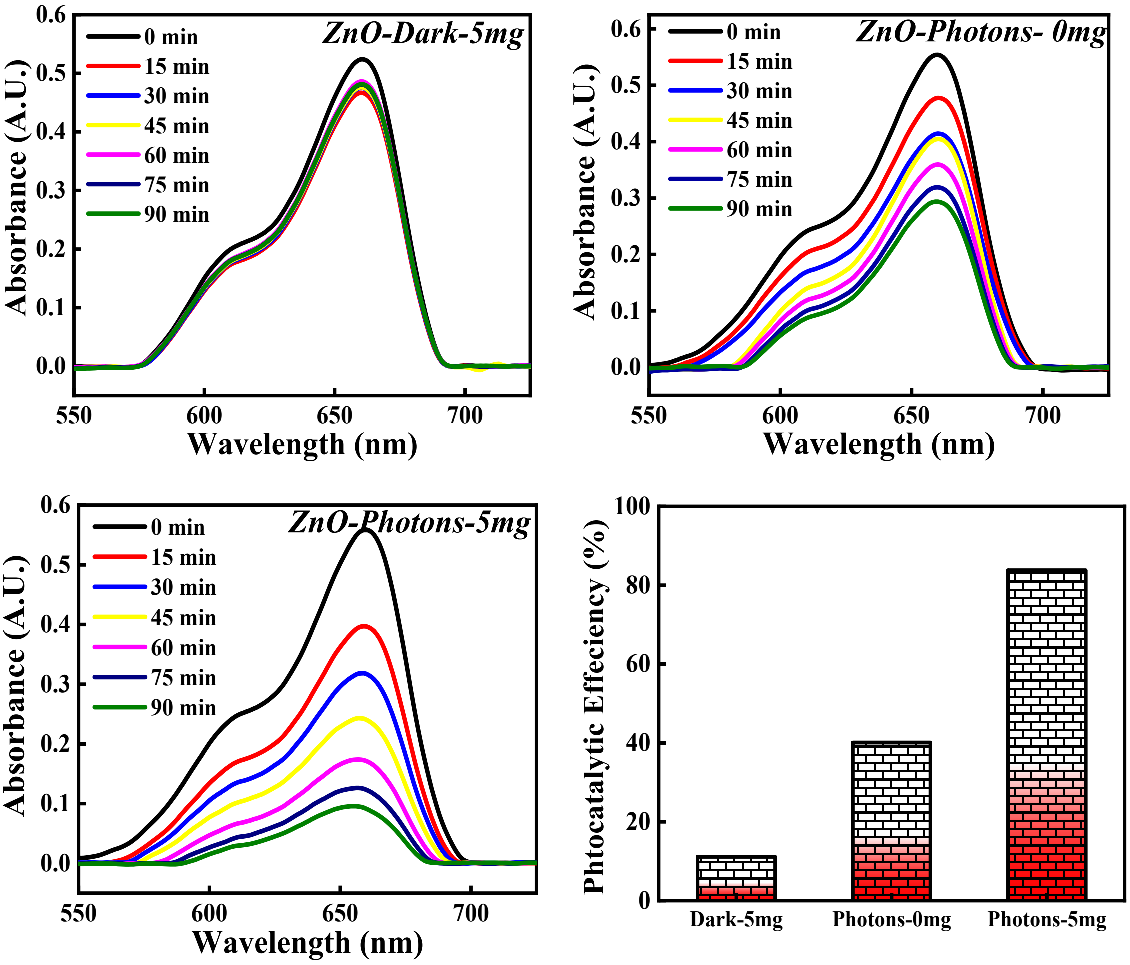

Figure S8: The photodecomposition of MB dye based on dark reactions, sunlight reactions without and with the presence of catalyst.

The role of ZnO photocatalyst and sunlight in determining the photodegradation of MB dyewere explored based on dark reaction with 5 mg ZnO catalyst, sunlight reaction without and with 5mg ZnO catalyst. The obtained photocatalytic degradation plots are represented in figure 19. It is noticed that the percentage of photocatalytic decomposition of MB dye with respect to dark reaction containing catalyst is 13.05 %, sunlight reactions without and with catalyst is 40.10 % and 84 % respectively. It is interesting to see that the sunlight reaction without catalyst has shown 40.10 % degradation of MB dye but it is not always true (as seen in the decrement in the absorbance values). This is because, even though MB absorbance values at 663 nm is decreasing simultaneously but it doesn't mean that dye degradation is

enhancing. The MB^+ is still there which can be observed at 284 nm as shown in figure S9. Moreover, there is LMB formation whose characteristic peak is at 245 nm[54]as shown in figure S10. The MB^+ and LMB formation is not seen in sunlight reaction with 5mg catalyst. So, the decrease in absorbance values at 663 nm for sunlight reaction without catalyst is not the proof towards the degradation of dye. The dye degradation of MB dye is only valid until it's all intermediates is vanished completely. The 13.05 % degradation of MB dye for the dark reactions can be due to the adsorption of MB dye molecules on the catalyst surface whereas 84% MB degradation for 5mg ZnO catalyst in sunlight is due to the generation of e-h pairs, radical generation, adsorption of dye molecules etc which has discussed in earlier part. Hence, for the effective decomposition of MB dye, both sunlight and ZnO catalyst play a crucial role.

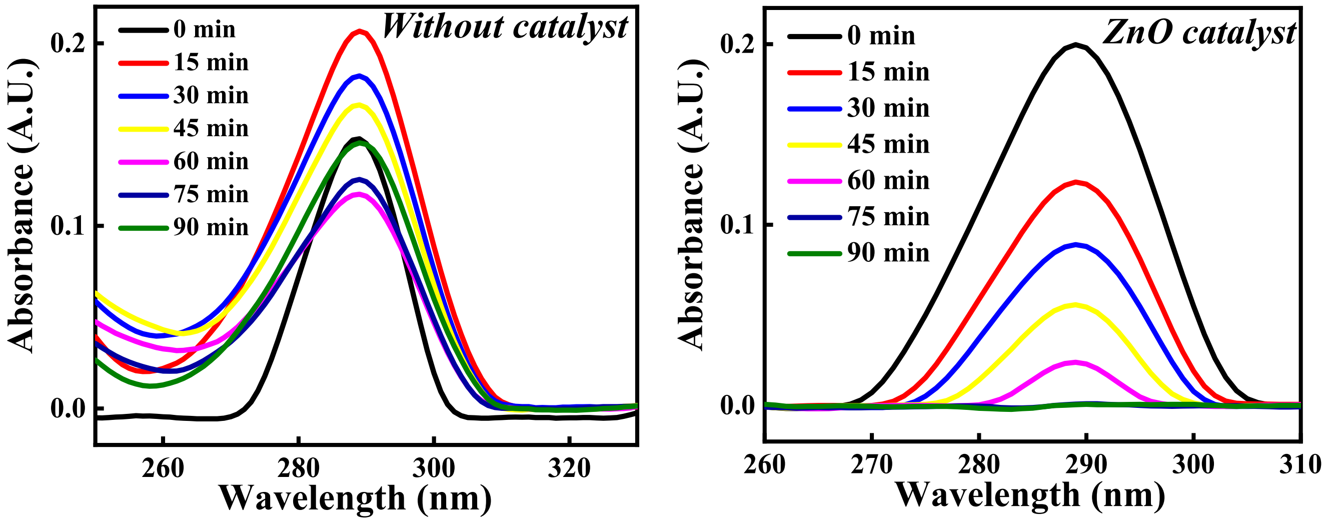

Figure S9: The photodegradation of MB^+ dye based on sunlight reaction without and with the presence of catalyst

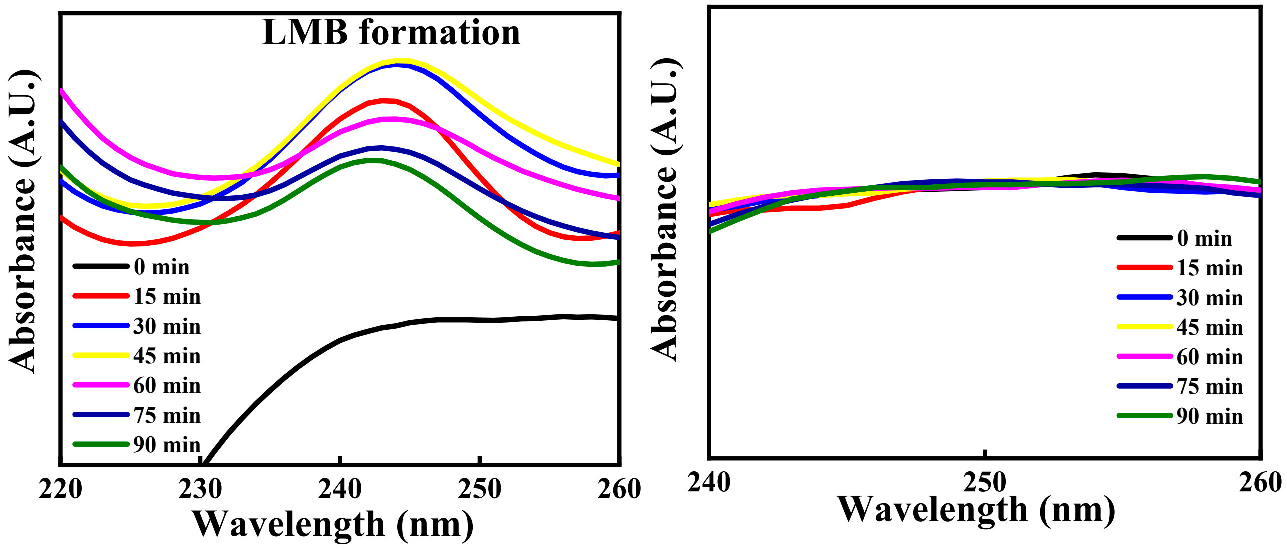

Figure S10: (Left) LMB formation in sunlight reaction without catalyst, (Right) No LMB formation in sunlight reaction with catalyst.

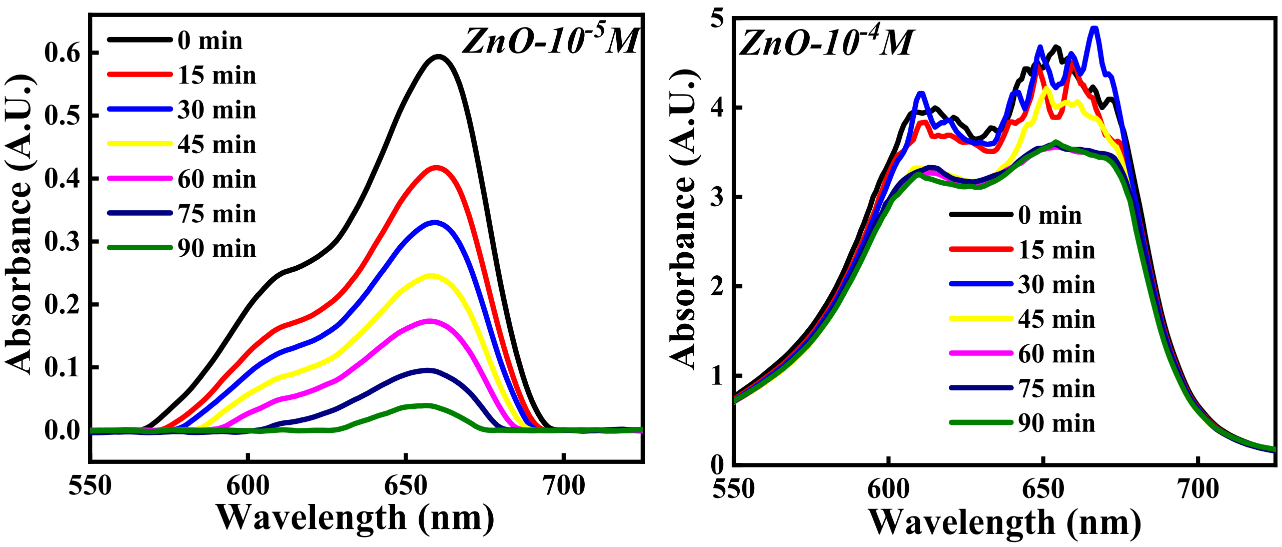

Figure S11: The photocatalytic activity of ZnO based on increase in the concentration of MB dye.
